# Resistance to checkpoint blockade therapy through inactivation of antigen presentation

**DOI:** 10.1038/s41467-017-01062-w

**Published:** 2017-10-26

**Authors:** Moshe Sade-Feldman, Yunxin J. Jiao, Jonathan H. Chen, Michael S. Rooney, Michal Barzily-Rokni, Jean-Pierre Eliane, Stacey L. Bjorgaard, Marc R. Hammond, Hans Vitzthum, Shauna M. Blackmon, Dennie T. Frederick, Mehlika Hazar-Rethinam, Brandon A. Nadres, Emily E. Van Seventer, Sachet A. Shukla, Keren Yizhak, John P. Ray, Daniel Rosebrock, Dimitri Livitz, Viktor Adalsteinsson, Gad Getz, Lyn M. Duncan, Bo Li, Ryan B. Corcoran, Donald P. Lawrence, Anat Stemmer-Rachamimov, Genevieve M. Boland, Dan A. Landau, Keith T. Flaherty, Ryan J. Sullivan, Nir Hacohen

**Affiliations:** 1 Department of Medicine, Massachusetts General Hospital Cancer Center, Boston, MA 02114 USA; 2 Broad Institute of the Massachusetts Institute of Technology (MIT) and Harvard, Cambridge, MA 02142 USA; 3 Department Systems Biology, Harvard Medical School, Boston, MA 02115 USA; 4 Department of Pathology, Massachusetts General Hospital, Boston, MA 02114 USA; 5 Department of Medical Oncology, Dana-Farber Cancer Institute, Boston, MA 02215 USA; 6 Department of Biostatistics and Computational Biology, Dana-Farber Cancer Institute, Boston, MA 02215 USA; 7 Department of Surgery, Massachusetts General Hospital, Boston, MA 02114 USA; 8 New York Genome Center, NYC, New York, NY 10013 USA; 9 Department of Medicine and Department of Physiology and Biophysics, Weill Cornell Medicine, NYC, New York, NY 10065 USA

## Abstract

Treatment with immune checkpoint blockade (CPB) therapies often leads to prolonged responses in patients with metastatic melanoma, but the common mechanisms of primary and acquired resistance to these agents remain incompletely characterized and have yet to be validated in large cohorts. By analyzing longitudinal tumor biopsies from 17 metastatic melanoma patients treated with CPB therapies, we observed point mutations, deletions or loss of heterozygosity (LOH) in beta-2-microglobulin (*B2M*), an essential component of MHC class I antigen presentation, in 29.4% of patients with progressing disease. In two independent cohorts of melanoma patients treated with anti-CTLA4 and anti-PD1, respectively, we find that *B2M* LOH is enriched threefold in non-responders (~30%) compared to responders (~10%) and associated with poorer overall survival. Loss of both copies of *B2M* is found only in non-responders. *B2M* loss is likely a common mechanism of resistance to therapies targeting CTLA4 or PD1.

## Abstract (web summary)

Resistance to immune-checkpoint blockade often occurs in treated patients. Here, the authors demonstrate that *B2M* loss is a mechanism of primary and acquired resistance to therapies targeting CTLA4 or PD-1 in melanoma patients.

## Introduction

Although immune checkpoint blockade (CPB) with ipilimumab (anti-CTLA4), pembrolizumab and nivolumab (anti-PD1), and atezolizumab (anti-PDL-1), leads to prolonged responses in 15–40% of patients with metastatic melanoma, treatment refractory disease, and progression after initial response remain major causes of mortality^1–3^. Checkpoint inhibitors are designed to unleash tumor-specific T-cell immunity by targeting co-inhibitory receptors and their ligands, and have been approved by the FDA for the treatment of metastatic melanoma^4, 5^ and other types of solid tumors^6^. More specifically, the efficiency of CPB depends on cytotoxic CD8^+^ T-cell (CTL) recognition of cancer-specific antigens presented on human leukocyte antigen (HLA) class I complexes, which are composed of a heavy-chain and beta-2-microglobulin (B2M), a crucial factor required for to the assembly of all HLA class I complexes and for the stable presentation of antigens by the tumor cells^7^.

A deeper understanding of the mechanisms underlying response and resistance to CPB, coupled with more effective predictive biomarkers, would enable earlier detection of progressive disease and inspire new therapeutic strategies that induce long-term responses. To date, several clinical predictors of CPB response in melanoma have been identified (e.g., mutational and neoantigen loads, PDL-1 expression), with PDL-1 expression being used in practice to select patients for therapy^8–10^. In pretreatment tumor samples, defects in the IFN γ pathway^11^ were identified as a mechanism of primary resistance to anti-CTLA4 therapy. Moreover, in post-treatment samples, mutations in *JAK1*, *JAK2*, and *B2M* were recently proposed as mechanisms of acquired resistance in three melanoma patients treated with anti-PD-1^12^. Despite these advances, the basis for lack of response and resistance to different CPB remains unknown in most patients and has not been studied in large cohorts. We hypothesized that the strong immune pressure imposed by different CPB therapies on tumor populations could lead to the selection of common mechanisms of resistance, enabling the tumor to evade immune destruction.

To find common mechanisms of intrinsic and acquired resistance to CPB therapy, we analyzed somatic genetic alterations in longitudinal samples in a cohort of patients treated with several CPB and validated our results in two large cohorts. While we find no significant changes in both mutational and neoantigen loads over time between responders and non-responders, we identify *B2M* aberrations in 29.4% of patients with progressing disease, including multiple early frameshift mutations, LOH overlapping *B2M*, and absence of tumor-specific B2M protein expression. Additional defects in the antigen presentation and IFNγ pathways are identified but are not restricted to progressing lesions in our cohort. In two independent cohorts of 105 and 38 melanoma patients treated with ipilimumab (anti-CTLA4) and pembrolizumab (anti-PD1), respectively, we find that *B2M* LOH is enriched threefold in non-responders (~30%) vs. responders (~10%) and associated with poorer overall survival (log-rank *p* = 0.01, *p* = 0.006). Loss of both copies of *B2M* is found only in non-responders. We also find evidence for association of LOH overlapping *IFNGR1* with poorer overall survival exclusively in the anti-PD1 cohort. Overall, these results imply that *B2M* loss is a common mechanism of intrinsic and acquired resistance to CPB inhibitors, and should stimulate development of new therapeutic strategies.

## Results

### Genetic analysis of longitudinal biopsies

To dissect the genetic alterations associated with intrinsic and acquired resistance, we performed WES on 49 longitudinal tumor (and matched blood) samples from 17 patients with metastatic melanoma, 10 of whom initially responded to CPB, with 5 having durable responses and 5 who eventually progressed (Supplementary Data 1 and Supplementary Fig. 1). Frequency of cancer cells with mutations in 22 known melanoma drivers remains relatively constant over time, suggesting minimal contribution to CPB escape^13^ (Supplementary Fig. 2). While a positive correlation between objective response and survival was observed in our cohort, genome-wide analysis showed no significant differences in the overall mutation load (Supplementary Fig. 3) or neoantigen load (Supplementary Figs. 4 and 5) over time or between responders and non-responders, consistent with previously observed weak associations^8, 14^. Similarly, we saw no significant difference in somatic mutation loads in genes related to the HLA presentation (Supplementary Fig. 6) or interferon-gamma pathways (Supplementary Fig. 7), which have been recently implicated as both primary and acquired resistance mechanisms to CPB^11, 12^. The lack of a positive correlation between response and somatic mutation burden in these two pathways could be due to the limited cohort size or to aggregation of all mutations, thus masking a subset of mutations associated with clinical outcome.

### Acquired resistance through the loss of *B2M*

**Fig. 1:**
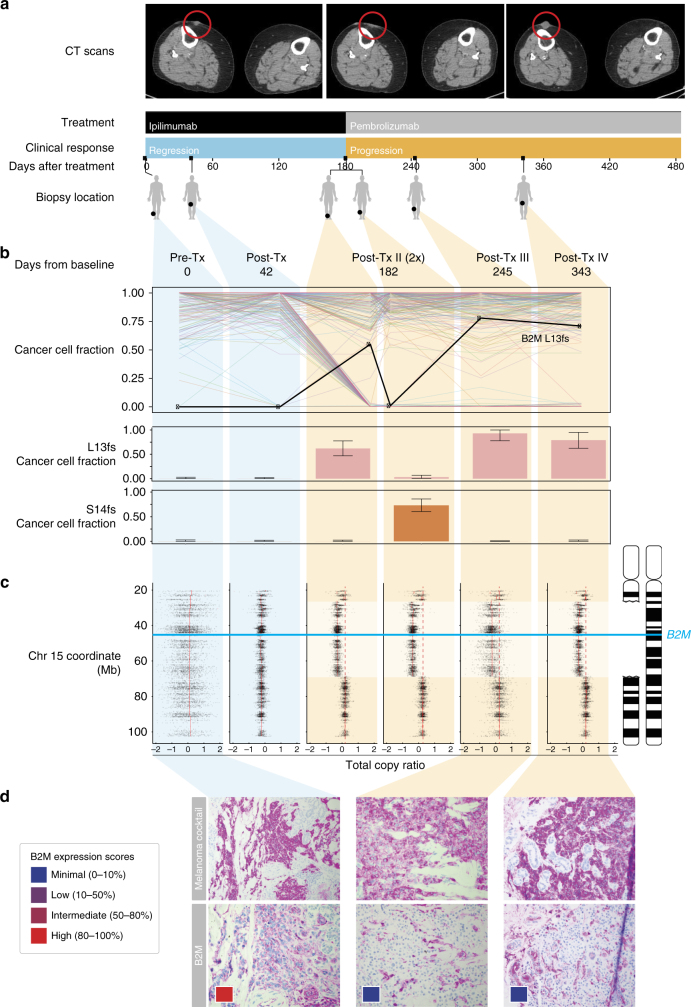
Loss of *B2M* is associated with resistance in a patient treated with checkpoint blockade. **a** Treatment and sample collection timeline for Pat208. Row 1, computed tomographic (CT) images of right thigh taken at baseline, during response and relapse; row 2, CPB treatments (ipilimumab—anti-CTLA4, pembrolizumab—anti-PD1); row 3, clinical response while on treatment, with blue indicating regression and orange indicating progression; row 4, days elapsed with respect to the start of treatment; row 5, location of biopsies taken at the different time points. **b** Criteria used to identify potential drivers of resistance, were genes with multiple non-silent mutations and LOH that are dominant only during disease progression. Out of 248 mutations with adequate coverage across all samples (detection power ⩾0.9), only *B2M* mutations satisfied all criteria (upper panel). Fraction of cancer cells harboring two separate early frameshift mutations in *B2M* (p.Leu13fs and p.Ser14fs) detected in Pat208. Blue backgrounds indicate samples taken during disease regression, and orange backgrounds indicate samples taken during disease progression. Error bars indicate 95% confidence intervals as inferred by ABSOLUTE (lower panel, described in “Methods” section). **c** Illustration of the deletions locations on chromosome 15 overlapping the *B2M* locus found in Pat208, as well as the location of the two early frameshift mutations relative to the *B2M* gene (blue line); and the total copy ratios of target regions on chromosome 15 in each biopsy. Red dashed lines indicate an absolute total copy number of 2 as inferred by ABSOLUTE. A deleted region overlapping the *B2M* locus is seen in all relapse samples (light orange background). **d** Samples were stained with an antibody cocktail for melanoma cells (mel.cocktail) using anti-melanosome (HMB45), anti-MART-1/melan A and anti-Tyrosinase, to discern melanoma cells from normal cells; or with an antibody specific for B2M. Colored boxes indicate B2M expression scores: B2M scoring was estimated by using four different levels of expression in the tumor fraction: minimal, 0–10%; low, 10–50%; intermediate, 50–80%; and high, 80–100%, B2M expression in the tumor fraction. Original magnification ×100

As the genetic landscape over the entire cohort was insufficient to explain patient outcome, we next performed a focused case study. From the five patients with initial response followed by disease progression, we focused on Pat208 who showed a response lasting more than 6 months and had a total of six high-quality biopsies at baseline, disease regression, and disease progression (Fig. 1a). To identify potential drivers of resistance in Pat208, we looked for genes showing mutations concurrent with loss of heterozygosity (LOH) that were present at high cancer cell fractions only during progressive disease but not while the patient was responding. Of 248 mutations with adequate coverage across all samples (detection power ≥0.9) (Supplementary Data 2), only *B2M* mutations satisfied all criteria (Fig. 1b). Specifically, we found two frameshift mutations in exon-1 of *B2M*: p.Leu13fs and p.Ser14fs and confirmed the presence of these mutations only during progression by targeted Sanger sequencing and manual review of sequenced reads at the *B2M* locus (Supplementary Figs. 8 and 9). In the same patient, chromosome 15 segment copy ratios show similar breakpoints in all progression samples, indicating a single *B2M* deletion event preceding the acquisition of p.Leu13fs and p.Ser14fs (Fig. 1c). Due to this deletion/LOH and upstream frameshift mutations in *B2M*, the majority of cancer cells in progression samples were likely to be *B2M* deficient.

The spatial distribution and population frequency of the two *B2M* mutations suggest that a tumor lineage diverged early, developed *B2M* LOH, and branched into two separate CPB-resistant populations, each with a distinct early frameshift in *B2M*. Despite the spatial proximity of post-Tx-II-1 and post-Tx-II-2 (Fig. 1a), p.Ser14fs was only detected in post-Tx-II-2 (74% of cancer cells) and not post-Tx-1 (0%). In contrast, p.Leu13fs was high only in post-Tx-II-1 (55%) but not in post-Tx-II-2 (1%) (Fig. 1b and Supplementary Fig. 9). Phylogenetic reconstruction using all mutations found in Pat208 biopsies resulted in two major lineages branching early in the tumor’s evolutionary history. Biopsies taken before progressive disease were composed of lineages on the left branch, while biopsies taken after progressive disease were composed of lineages on the right branch (Supplementary Fig. 10). While this pattern could be attributed to spatial heterogeneity of lesions, the absence of lineages from the left branch of the tree in all four progression biopsies and the dominance of new *B2M*-deficient lineages suggest that *B2M* loss led to significant selective advantages. These results support the idea of immunoediting^15^ as a mechanism of resistance to CPB, were the “immunogenic” tumor cells have been edited by the immune system, enabling the *B2M*-deficient (“non-immunogenic”) clones to evade immune destruction.

To explore whether genetic alterations in *B2M* led to loss of protein expression, immunohistochemistry (IHC) was performed on Pat208 biopsies. As expected from the identified *B2M* frameshift mutations, a dramatic drop in tumor-specific B2M protein levels occurred after Pat208-developed resistance (Fig. 1d). Since *B2M* is essential to the assembly of all HLA class I complexes and for the stable presentation on the cell surface^7^, we next examined HLA class I expression by staining biopsy samples with pan-HLA class I antibodies in Pat208. While HLA class I proteins localized appropriately to the outer cell membrane of melanoma cells during response to CPB, they were not detected on the outer membrane during disease progression (Supplementary Fig. 11a). The presence of simultaneous LOH and frameshift *B2M* mutations in Pat208, along with concurrent loss of B2M and HLA Class I protein expression, suggests that *B2M* aberrations could contribute to tumor evasion of CD8^+^ T-cell responses and disease progression.

### Changes in the tumor microenvironment during relapse

**Fig. 2:**
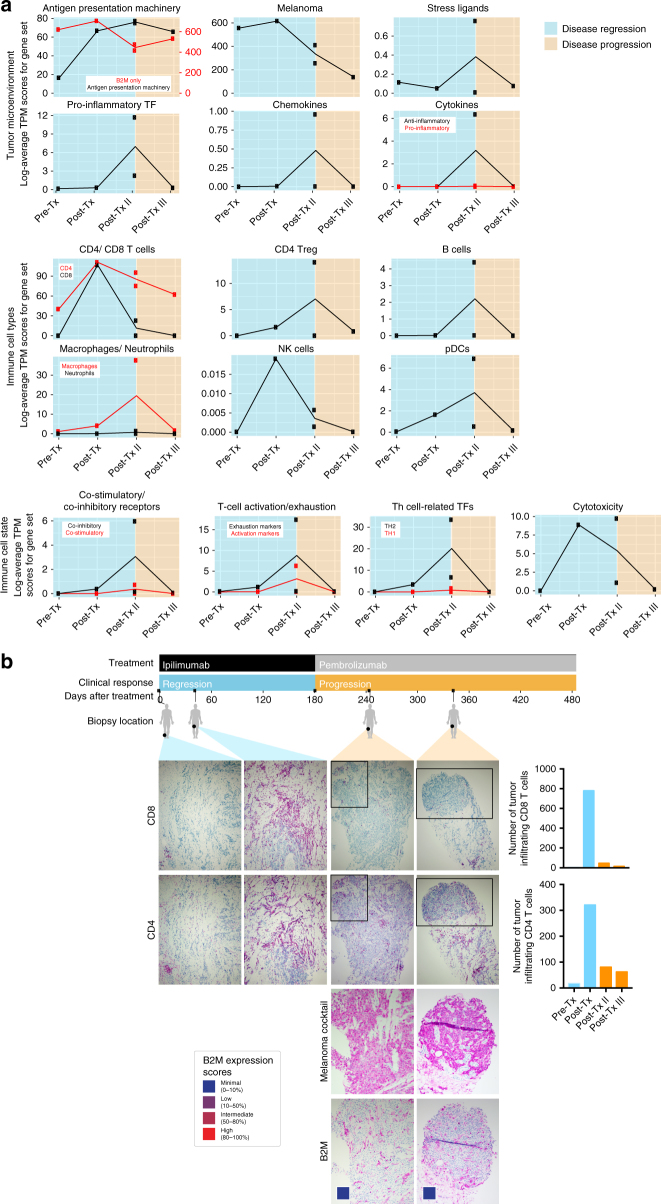
Dissecting the tumor microenvironment during disease regression and progression. **a** Expression scores of genes related to the tumor microenvironment, immune cell types, or immune cell states. Expression scores were calculated as the geometric mean of TPM values of genes in Supplementary Data 3. Blue backgrounds indicate biopsies taken during disease regression, and orange backgrounds indicate biopsies taken during disease progression. **b** Samples for each time point from Pat208 were stained with specific antibodies against CD8 or CD4 and were quantified using the cell counter function in Fiji (described in “Methods” section). Boxes areas in the upper panels (CD4 and CD8, original magnification ×100) are shown at higher magnification (×200) in the lower panels (melanoma cocktail and B2M). Colored boxes indicate B2M expression scores: B2M scoring was estimated by using four different levels of expression in the tumor fraction: minimal, 0–10%; low, 10–50%; intermediate, 50–80%; and high, 80–100%, B2M expression in the tumor fraction. A timeline of treatment, clinical response, and biopsy locations is shown at the top

Because checkpoint therapy depends on CTL recognition of tumor antigens presented on HLA class I proteins^4, 16^, we monitored changes in the immune cell infiltrate of tumors from Pat208. Based on RNA sequencing of bulk tumor biopsies (Fig. 2a and Supplementary Data 3), transcript levels of genes involved in antigen presentation, co-stimulation, inflammation, cytotoxicity, and CD8^+^ and NK cells increased during regression and subsequently decreased with progression. In agreement with bulk expression data, staining of biopsy sections (Fig. 2b) showed a dramatic decrease in CD8^+^ TILs, and to a lesser extent CD4^+^ TILs, during progressive disease, suggesting that, non-inflamed *B2M*-deficient tumors are less susceptible to infiltration by cytotoxic T cells.

### B2M defects in additional patients with progressing disease

Several other patients in our cohort exhibited *B2M* alterations. We discovered two *B2M* frameshift mutations, p.Ser14fs (50–90% of cancer cells) and p.Gly63fs (70–90% of cancer cells), in progression samples of PatT33, who initially responded for 1 year to ipilimumab. At baseline, p.Gly63fs was detected in 1/429 reads, and p.Ser14fs was undetectable (0/475); however, due to the low tumor purity of this baseline sample, no conclusions can be drawn about how these two mutations evolve with progression.

The presence of multiple frameshift mutations in *B2M* found in Pat208 and PatT33 suggested that this might be a mutation hotspot. Indeed, TCGA mutation data showed a cluster of *B2M* mutations at Ser14 (Supplementary Fig. 12a). All *B2M* mutations found in this cohort lie within 4× dinucleotide repeats (Supplementary Fig. 12b), some of which were seen in high-level microsatellite instability colorectal cancers^17, 18^, thus implicating faulty DNA mismatch repair (MMR). No DNA mutations in MMR (*MLH1*, *MSH2*, *MSH3*, *MSH6*, and *PMS2*) were found in Pat208. A somatic mutation in *MSH2*, p.P476S, was found in post-Tx and post-Tx-II samples of PatT33.

**Fig. 3:**
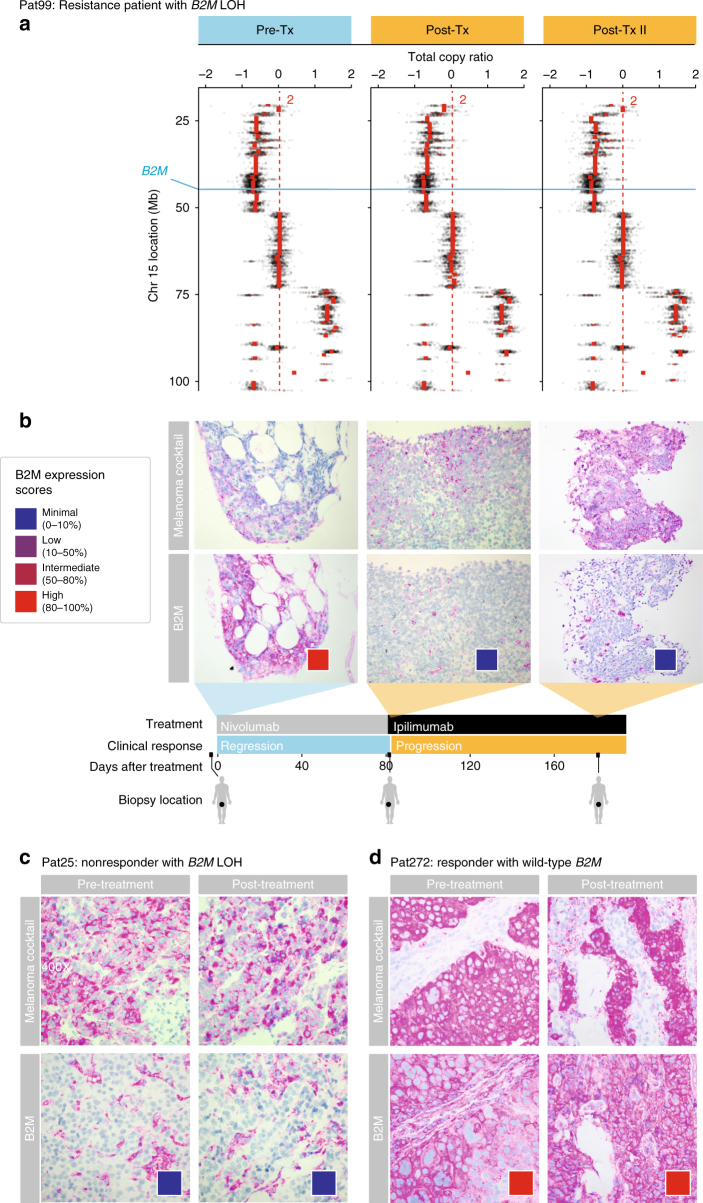
LOH in *B2M* is found in resistance and non-responding patients treated with CPB. **a** Illustration of the deletion locations on chromosome 15 overlapping the *B2M* locus found in Pat99 (blue line); and the total copy ratios of target regions on chromosome 15 in each biopsy. Red dashed lines indicate an absolute total copy number of 2 as inferred by ABSOLUTE. A deleted region overlapping the *B2M* locus is seen in all samples. Top row indicates the timeline of treatment (blue–regression, orange–progression). **b**–**d** Samples from Pat99 (**b**), Pat25 (**c**), and Pat272 (**d**) were stained with an antibody cocktail for melanoma cells (mel.cocktail) using anti-melanosome (HMB45), anti-MART-1/melan A and anti-Tyrosinase, to discern melanoma cells from normal cells; or with an antibody specific for B2M. Colored boxes indicate B2M expression scores: B2M scoring was estimated by using four different levels of expression in the tumor fraction: minimal, 0–10%; low, 10–50%; intermediate, 50–80%; and high, 80–100%, B2M expression in the tumor fraction. Original magnification ×100

In addition to *B2M* mutations found in Pat208 and PatT33, LOH that includes *B2M* was observed in all samples of Pat99, a patient who progressed after a brief 2.5-month period of regression following treatment with nivolumab (anti-PD1) (Fig. 3a). Tissue staining showed loss of tumor-specific B2M protein expression during and after progressive disease, but not while the patient was responding (Fig. 3b). Similar to Pat208, tissue staining for CD4^+^ and CD8^+^ T cells in Pat99 (Supplementary Fig. 13) showed a dramatic decrease in CD8^+^ TILs, and to a lesser extent CD4^+^ cells, during progressive disease after *B2M* loss but not during response. Two more non-responders, Pat25 and Pat115, had LOH that includes *B2M* (Supplementary Fig. 14). In contrast to Pat99, loss of tumor-specific B2M protein expression was found in all samples from Pat25 (Fig. 3c). In addition, in Pat99 and Pat25, HLA class I was localized to the cytosol during disease progression (Supplementary Fig. 11). No slides were available for Pat115. Validation of B2M expression in a responder (Pat272) showed no changes in tumor-specific expression over time (Fig. 3d). We conclude that in addition to genetic mutations leading to loss of *B2M* (e.g., Pat208), other non-genetic mechanisms also use by tumor cells (e.g., Pat99 and Pat25) and may lead to transient or long-term reduction in antigen presentation.

Collectively, 5 out of 17 patients (29.4%) in our cohort exhibited *B2M* defects, with three of five patients who initially responded and then progressed (Pat208 with LOH and FS mutation; Pat33 with FS mutations; Pat99 with LOH), and two of seven non-responders (Pat25 and Pat115 with LOH) (Supplementary Data 1). No *B2M* alterations were detected in responders within our cohort. We also found that sequencing of cell-free DNA isolated from blood samples detected *B2M* frameshifts in Pat208 and *B2M* LOH in Pat99 (Supplementary Fig. 15). Although sequencing cell-free DNA from blood samples may not be sensitive enough when tumor burden is low, it does provide a minimally invasive way of monitoring resistance mutations for patients receiving CPB.

### Defects in the antigen presentation and IFNγ pathways

Since *JAK1*, *JAK2*, and *IFNGR1* were recently implicated as drivers of acquired or primary resistance to anti-PD1 and anti-CTLA4 therapies, respectively^11, 12^, we looked at the frequency of mutations and LOH in these genes within our cohort. We found six *JAK1* alterations, including a missense mutation in one non-responder, and LOH in five more patients (two non-responders, two resistant patients, and one responder), including PatT33, who developed resistance to CPB and also had two *B2M* frameshift mutations. LOH overlapping *JAK2* was detected in 10 samples (4 non-responders, 3 resistance patients, and 3 responders), and LOH overlapping *IFNGR1* was detected in 5 samples (3 non-responders, 1 resistant patient, and 1 responder) (Supplementary Data. 4). We did not detect any mutations in *JAK2*, *IFNGR1*, and *IFNGR2* within our 17 patient cohort, but identified alterations in other genes related to the antigen presentation and IFNγ machineries (e.g., *STAT1*, *STAT2*, *TAP1*, and *TAP2*), which were not restricted to progressing samples. Although only *B2M* alterations were exclusively present in non-responders within our cohort, the mutations observed in the interferon pathway may also contribute to immune evasion by tumor populations in response to CPB.

### Detection of *B2M* aberrations in two independent cohorts

**Fig. 4:**
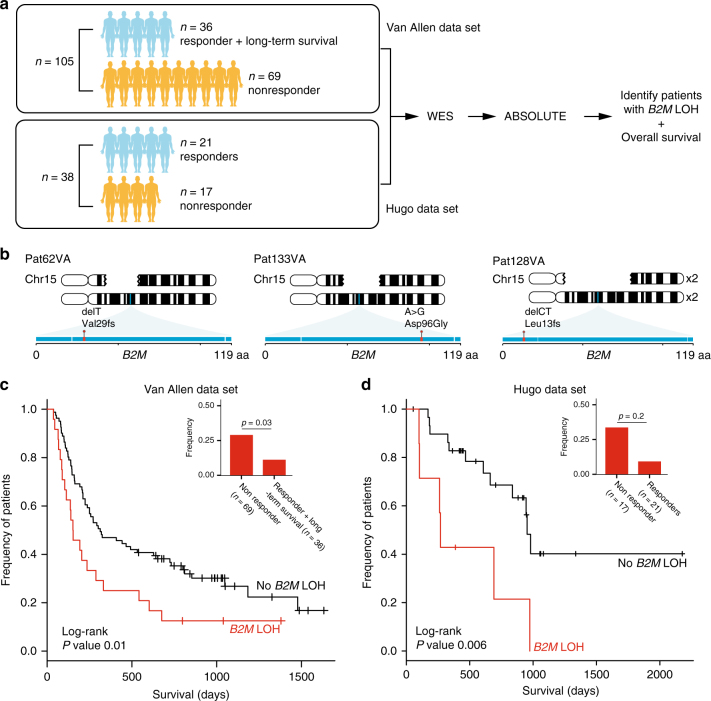
Clinical relevance of *B2M* aberrations in two independent cohorts. **a** Analysis workflow for both Van Allen (105 patients pre-anti-CTLA4 treatment) and Hugo (38 patients pre-anti-PD1 treatment) data sets. For both data sets, we analyzed whole exome sequences of paired tumor and normal biopsies using the same pipeline used to analyze our cohort. **b** Illustration of three non-responders in the Van Allen data set with nucleotide mutations in *B2M* accompanied by loss of the wild-type allele. Gaps in the top chromosome depict the deleted region in each patient. Exons in *B2M* are shown as a horizontal blue rectangle, with mutations found in each patient highlighted in red. **c** Kaplan–Meier survival curves for patients in the Van Allen data set with (red) and without (black) *B2M* LOH. Log-rank *p* value is shown (*p* < 0.01). Inset shows the frequency of patients with *B2M* LOH in non-responders vs. responders and long-term survivors. One-sided Fisher’s exact *p* value is shown (*p* < 0.03). **d** Identical analysis performed for the Hugo data set as in **c**

Next, we validated the clinical importance of *B2M* aberrations in two independent cohorts. In the first cohort composed of biopsies from 110 patients prior to anti-CTLA4 therapy^8^, we found *B2M* aberrations to be a significantly enriched in non-responders and significantly associated with poorer survival. After filtering out five biopsies with low tumor content, this data set was composed of 26 responders, 69 non-responders, and 10 patients who were defined as long-term survivors with no objective clinical response (Fig. 4a, Van Allen data set). Two frameshift and one missense *B2M* mutations, including p.Leu13fs, were discovered in three non-responders, but not in any responders (Fig. 4b). In all three, *B2M* LOH also occurred, leading to complete loss of *B2M*. As our data implicated LOH as a more frequent form of *B2M* alteration and a potential precursor to the loss of B2M protein expression, we investigated the presence of *B2M* LOH in this large cohort. *B2M* LOH events were significantly enriched in non-responders (20/69, 28.9% vs. 4/36, 11.1%, one-sided Fisher’s exact *p* = 0.03), and significantly associated with poorer overall survival (log-rank *p* = 0.01) (Fig. 4c). Similarly, in the second cohort composed of biopsies from 21 responders and 17 non-responders (*n* = 38) before anti-PD1 treatment^14^ (Fig. 4a, Hugo data set), we found *B2M* LOH to be significantly associated with a worse overall survival (log-rank *p* = 0.006) (Fig. 4d). *B2M* LOH was not significantly enriched (Fisher’s exact test), although the proportion of patients with *B2M* LOH was very similar to our and the Van Allen cohort (non-responders 5/17, 29.4% vs. responders 2/21, 9.5%) (Fig. 4d). No nucleotide mutations were found in this data set, consistent with the frequency at which *B2M* nucleotide mutations were seen in the Van Allen data set. Unlike *B2M*, LOH in genes involved in the IFNγ and antigen presentation pathway were not significantly enriched in non-responders, and did not significantly associate with poorer overall survival across both data sets, except for LOH in *IFNGR1* that was significantly associated with lower overall survival only in the Hugo data set (Supplementary Data 4 and Supplementary Fig. 16). In contrast to *B2M*, mutations in *IFNGR1*, *JAK1*, *JAK2*, *STAT2*, and *TAP1/2* were found in both non-responders and responders in these two independent cohorts. Given the enrichment of *B2M* aberrations in non-responders and their significant association with lower overall survival, and given the validation of these findings in three cohorts treated with different CPB therapies, we suggest that LOH and mutations in *B2M* contribute to a common mechanism of resistance to various CPB therapies.

### NK cell control the expansion of *B2M*-deficient clones

Finally, an important clinical question is whether *B2M*-deficient melanomas can be targeted with NK cells, given the known inhibitory effect of HLA class I proteins on NK cells^19, 20^. We thus developed a mouse model to test whether natural killer cells would selectively kill *B2M*-deficient melanoma cells. Using the CRISPR/Cas9 technology, we introduced a guide RNA and deleted *B2M* in a melanoma cell line derived from *BRAF* ^V600E^; *PTEN* ^−/−^ transgenic mice (Supplementary Fig. 17a, b). To mimic evolving human tumors, we transplanted a mixture of the parental *B2M* ^*+/+*^ and the *B2M*-deleted cell lines (each tagged with unique markers) into mice in the presence or absence of NK cells (using antibodies to deplete NK cells) (Supplementary Fig. 17c). Mice that lacked NK cells showed a significant increase in *B2M*-deleted relative to *B2M* ^+/+^ tumor cells (Supplementary Fig. 17d). Thus, NK cells offer a potential strategy to overcome resistance caused by loss of *B2M*.

## Discussion

Recent studies have identified several genetic drivers of primary or acquired resistance to CPB therapy, including one patient with an acquired *B2M* mutation, but were restricted to one type of therapy (either anti-CTLA4 or anti-PD1)^11, 12^. While non-LOH *B2M* aberrations are rare in melanoma (1.7–4% of cases)^13^, *B2M* functional loss has been hypothesized as a mechanism of immune escape in some cancers including melanoma^12, 17, 21, 22^. Furthermore, dinucleotide repeats in *B2M* become hotspots for frameshift indels, especially in tumors with aberrant MMR genes, as seen in high-level microsatellite instability colorectal cancers^23^. Our demonstration of *B2M* mutations and LOH only in progression samples in our cohort (several CPB therapies, *n* = 17 patients, 49 longitudinal samples) as well as enrichment of *B2M* LOH in non-responders with poorer overall survival in two independent cohorts (anti-CTLA4, *n* = 105; anti-PD1, *n* = 38), suggests that *B2M*-mutated tumor subclones are selected through immunoediting earlier in tumor development, or under selective pressure applied by CPB. Although several genes are responsible for the processing, loading, and presentation of antigens, and have been shown to be mutated in cancers^24^, no proteins can substitute for *B2M* in HLA class I presentation, making the loss of *B2M* an evolutionary attractive route for CPB resistance. Additionally, the finding that *B2M* mutations exclusively are seen in pretreatment samples from patients who did not respond to CPB or in post-progression samples after initial response to CBP, whereas mutations in other genes, such as *JAK1*, *JAK2*, *IFNGR1*, *STAT2*, and *TAP1/2*, were seen in pretreatment samples from responders and non-responders alike, highlights the potential importance of *B2M* in CPB resistance. Finally, our data also implicated LOH as a more frequent form of *B2M* alterations in non-responders (as well as *IFNGR1* solely in the Hugo anti-PD1 cohort). LOH in *B2M* might be the initial event toward complete loss of the *B2M* gene, followed by MHC class I and antigen presentation loss. Indeed, it has been shown that LOH on chromosome 15 overlapping *B2M* is enriched in other types of cancer, such as breast, bladder, and MSS colon carcinomas (53, 44, and 35%, respectively)^25^.

Several important questions remain to be addressed based on these results. First, what is the role of epigenetic regulation in suppressing B2M expression when only one copy of the gene is missing or defective? Second, how frequently is the resistance, primary or acquired, to CPB therapy explained by *B2M* mutations and/or loss of B2M/HLA protein expression levels prior to or during treatment? Third, will therapies that target NK cell activation, such as the induction of NK-activating ligands on tumor cells by radiation or DNA-damaging agents^20^, improve outcomes of CPB by targeting resistant cells that are deficient for antigen presentation? As checkpoint blockade immunotherapy has become a mainstay of cancer therapy, it is imperative that future efforts build upon the work presented here to answer these questions and improve outcomes for our patients.

## Methods

### Patients samples

Patients with metastatic melanoma provided written informed consent for the collection of tissue and blood samples for research and genomic profiling, as approved by the Dana-Farber/Harvard Cancer Center Institutional Review Board (DF/HCC Protocol 11–181). Matched tumor and normal blood samples were obtained from 17 patients at baseline and after CPB treatment.

### WES sequencing

Whole exome sequencing of DNA extracted from fresh frozen tumors and matched normal blood samples was done as previously described^8, 26^. All procedures were done at the Genomics Platform of the Broad Institute of Harvard and MIT.

DNA was extracted using Qiagen AllPrep DNA/RNA Mini Kit (cat# 80204) from fresh frozen tumor samples and stored at −80 °C. Germline DNA was extracted from matched peripheral mononuclear cells. An aliquot of 250–500 ng of DNA in 100 µl TE buffer was used as input for library generation. Palindromic forked adapters (Integrated DNA technologies) with unique 8 base index molecular barcode sequences were ligated to pool all samples. All other reagents used for end repair, A-base addition, adapter ligation, and library enrichment PCR were purchased from KAPA Biosciences in 96-reaction kits. After the post-library enrichment process, solid phase reversible immobilization (SPRI) beads cleanup (Beckman Coulter, cat# A63881) were used to reduce the volume to 20 µl to maximize library concentration. Library concentrations were measured by an automated PicoGreen assay on an Agilent Bravo instrument. All libraries above 40 ng/µl were considered acceptable for solution-phase hybrid selection and sequencing.

Samples were hybridized using Agilent SureSelect Human All Exon Kit v2 as previously described^27^. Samples were denatured at 95 °C, 5 min, then incubated at 65 °C, 17 h. DNA–RNA complexes were captured using the Agilent Bravo instrument. The reaction was carried out using the SureSelect Target Enrichment System Sequencing Platform Library Prep v2 (Agilent Technologies, cat# G3360-90000), according to the manufacturer’s specifications.

Libraries were quantified and normalized using PicoGreen to ensure equal concentration using a Perkin Elmer MiniJanus instrument and pooled by equal volume on the Agilent Bravo instrument. Library pools were quantified using quantitative PCR (KAPA Biosystems, cat# KK4832) with adapter-specific probes. After qPCR, libraries were brought to 2 nM and denatured using 0.2 M NaOH on the Perkin Elmer MiniJanus. After denaturation, libraries were diluted to 20 pM using hybridization buffer (Illumina).

Cluster amplification was performed according to the manufacturer’s protocol (Illumina), HiSeq 2500 v4 cluster chemistry and flowcells, as well as Illumina’s Multiplexing Sequencing Primer Kit. Libraries were sequenced using the HiSeq 2500 v4 Sequencing-by-Synthesis method (paired end 76 bp reads) followed by analysis with RTA v.1.12.4.2. The minimum depth of coverage was 150× and 80× for tumor and normal samples, respectively.

### Whole transcriptome sequencing

Whole transcriptome sequencing was performed as previously described^8^ using the Transcriptome Capture method. RNA was extracted using Qiagen AllPrep DNA/RNA Mini Kit (cat# 80204) from fresh frozen tumor samples and stored at −80 °C. An aliquot of 250 ng of purified total RNA was used with DV200 scores >30%. First a stranded cDNA library form isolated RNA was constructed followed by hybridization of the library to a set of DNA oligonucleotide probes to enrich the library for mRNA transcript fragments (capturing 21,415 genes, representing 98.3% of the RefSeq exome). The normalized, pooled libraries were loaded onto HiSeq 2500 for a target of 50 million 2 × 76 bp paired reads per sample.

### Gene expression analysis

Whole transcriptome data were processed on Cancer Genome Analysis tool “Firehose”. Alignment was performed using STAR^28^, and de-duplication using Picard. Transcripts per million (TPM) values were calculated using RSEM^29^. Expression scores in Fig. 2 were calculated as the geometric mean of TPM values of genes in Supplementary Data 3.

### Sanger sequencing

Genomic DNA extracted from Pat208 samples was used to validate the WES c.(37–39)ctcfs, p.Leu13fs; and c.(40–45)tctcttfs, p.Ser14fs mutations by using targeted Sanger sequencing. After DNA isolation, exon-1 in chromosome 15, where the two mutations are located was amplified using primers *B2M*_F (GGCATTCCTGAAGCTGACA) and *B2M*_R (GAAGTCACGGAGCGAGAGAG), followed by standard PCR conditions (95 °C 10 min; ×35 cycles (95 °C 30 s, 58 °C 15 s, 72 °C 15 s); 72 °C 5 min, 4 °C ∞), using Platinum PCR Supermix (Invitrogen, cat# 12532-016). Sanger sequencing was done using the *B2M*_R primer and was compared to normal control sample as a negative control.

### Analysis of whole exome sequencing

Whole exome sequencing data were processed sequentially via two pipelines. First, we used Picard (https://broadinstitute.github.io/picard/), a tool developed by the Genomics Platform at the Broad Institute, to process raw sequencing data from Illumina HiSeq. For each tumor or normal sample, Picard checks for contamination, aligns reads to hg19, and calculates quality metrics, resulting in a single de-multiplexed, aggregated BAM file (see http://samtools.github.io/hts-specs/SAMv1.pdf). After Picard, BAM files were processed using the Cancer Genome Analysis tool known as “Firehose”. Firehose takes paired BAM files from matched tumor and blood samples, and performs various functions, including quality control, local realignment, detection of somatic single-nucleotide variations (SSNVs), and somatic copy number alterations (SCNAs). Processing details involving Firehose have been detailed elsewhere (see http://www.broadinstitute.org/cancer/cga)^30^.

SSNVs, and insertions and deletions (INDELs) were called using a consensus voting algorithm that combined the outputs of Strelka^31^, VarDict^32^, and Mutect^33^. Only SSNVs and INDELs called by at least two callers were kept. For each, the union of all mutations found in each patient, reference, and alternate reads were counted at each variant locus with a custom “force-calling” script. SSNVs within coding regions of the genome were annotated for chromosomal location, variant type, genome change, codon change, and protein change using Oncotator^34^. All mutations called in *B2M* were manually verified in Integrative Genomics Viewer (IGV). Various quality controls were used to filter out artifacts due to formalin fixation or oxidation during library preparation. SCNAs were detected using Recapseg^34^, and allele-specific copy number variation was detected using AllelicCapseg. Both tools are available on Firehose.

Due to variable tumor fraction of biopsies, it is important to normalize the variant allele frequency, defined as the frequency at which a variant is seen out of the total number of reads at a position, to cancer cell fraction (CCF), the estimated fraction of cancer cells containing the variant. We used ABSOLUTE^35^ to infer CCF values for SSNVs and INDELs. In addition, ABSOLUTE also calculated ploidy, purity, and absolute DNA copy numbers of SCNAs. The power to detect events given sample purity and coverage was calculated by ABSOLUTE. Samples found to be non-aberrant by ABSOLUTE (SCNAs were absent and thus could not be used to determine optimal solutions during the manual curation step of ABSOLUTE) were excluded from analysis.

We used PhyloWGS^36^ to reconstruct complete genotypes and phylogenetic relationships of tumor subpopulations from CCF values of SSNVs, INDELs, and SCNAs. PhyloWGS is capable of performing on both WES as well as whole genome sequencing data (http://github.com/morrislab/phylowgs/issues/12). PhyloWGS separates variants into simple somatic mutations (SSMs) and copy number variations (CNVs). It corrects SSM frequencies in regions overlapping CNVs, and models CNVs as pseudo-SSMs. PhyloWGS is based on a generative probabilistic model. SSMS and CNVs are clustered using the non-parametric Dirichlet process prior. The clonal evolutionary structure is modeled with the tree-structured stick-breaking process prior. PhyloWGS then uses the Metropolis-Hastings algorithm, a Markov chain Monte Carlo procedure, to sample phylogenies from the model posterior that are consistent with SSM frequencies and evolutionary constraints.

For patients with more than 1000 mutational events, SSNVs and INDELs were clustered in PhyloWGS without SCNAs. CCF values were normalized by respective read depths and used as variant frequencies for input into PhyloWGS. Results for Pat208 required manual curation due to the PhyloWGS algorithm’s propensity for designating p.Leu13fs as the parent of p.Ser14fs, in violation of the “crossing rule” outlined in the PhyloWGS paper as well as the ample evidence for a sibling relationship between the two mutations. Thus, confidence for p.Ser14fs was artificially inflated by multiplying both the alternate and reference counts for the corresponding SSM by 1000. The resulting subtree rooted at the population containing p.Ser14fs was then merged back onto the original PhyloWGS output as a sibling of the subtree rooted at the population containing p.Leu13fs. All events assigned to the new subtree rooted at the population containing p.Ser14fs were then removed from other populations elsewhere in the tree such that no event was represented more than once.

### Calculation of neoantigen load

POLYSOLVER (POLYmorphic loci reSOLVER) was used to infer the HLA type for each patient, using sequencing data from the matched peripheral blood sample^37^. Potentially antigenic peptide sequences were inferred from mutational data. Neoantigen-binding predictions were made using NetMHCPan^38^. Peptides were designated as strong binders (mutant peptide with higher affinity than 0.5% of random natural peptides, with corresponding wild-type peptide having lower affinity than 2% of random natural peptides) or weak binders (mutant peptides with higher affinity than 2% of random natural peptides, with corresponding wild-type peptide having lower affinity than 2% of random natural peptides) with respect to the patient’s inferred HLA type. Mutated genes predicted to give rise to at least one neoantigen were used to calculate the neoantigen load.

### Visualization of TCGA data

Lollipop plots of *B2M* mutations found in TCGA data sets were visualized using cbioportal (http://www.cbioportal.org/), using the web query interface^39, 40^.

### Histology and staining

Immunohistochemistry staining was performed on four micrometer formalin-fixed paraffin-embedded sections. All procedures were done on the automated Ventana Discovery Ultra staining system. Sections were first deparaffinized with xylene and alcohol series, treated with EDTA retrieval solution and blocked with discovery inhibitor (Ventana products). Sections were incubated with primary antibodies for 16 min washed and incubated with a secondary antibody conjugated with horseradish peroxidase for additional 16 min. Discovery Purple Chromogen Kit (Ventana, cat# 760-229) was then applied to generate a color reaction. Slides were then counterstained with hematoxylin (Ventana). Primary antibodies used for staining were: anti-*B2M* (Abcam, cat# ab27588; 1:1000); anti-melanoma triple cocktail (HMB45 + A103 + T311; Ventana, cat# 790-4677; 1:100) containing the following antibodies: anti-Melanosome (HMB45), anti-MART-1/melan A (A103), and anti-Tyrosinase (T311); anti-CD8 (SP57; Ventana, cat# 790-4460; 1:150); anti-CD4 (SP35; Ventana, cat# 790-4423; 1:200), and anti-HLA class I ABC (EMR8-5; Abcam, cat# ab70328; 1:100). Protocols for all the staining are summarized in Supplementary Data 5. The anti-melanoma triple cocktail was used to discern melanoma cells from normal cells, allowing B2M, expression levels to be estimated for only the cancerous cell fraction. A single-blind scoring of cancer-specific B2M and tumor infiltrating CD4^+^, CD8^+^ T cells expression was conducted by two pathologists at the Massachusetts General Hospital. *B2M* scoring was estimated by using four different levels of expression in the tumor fraction: minimal, 0–10%; low, 10–50%; intermediate, 50–80%; and high, 80–100% *B2M* expression in the tumor fraction. Quantification of tumor infiltrating CD8^+^ and CD4^+^ T cells was evaluated manually using a 20× digital image of each case with the cell counter function in Fiji image processing software^41^.

### Analysis of independent cohorts

Sequencing data in the BAM format from tumor-normal pairs were analyzed using the identical pipeline for our cohort. In looking at the statistical significance of enrichment of *B2M* LOH in CPB non-responders vs. responders and long-term survivors, we determined that Fisher’s exact test was optimal due to the relatively small sample size and categorical nature of our data. Fisher’s exact test reports the exact *p* value under a null hypothesis of independent categories using the hypergeometric distribution. We determined the log-rank test, which is commonly used to compare survival data between cohorts, to be optimal for comparing the significance in survival differences between patients with and without LOH. The log-rank test assumes that data are right-censored, which are appropriate for our clinical data. The log-rank test further assumes a hypergeometric distribution of events (deaths) between patient groups at each time point. Here the assumption that events occur independently is reasonable. Here variance between groups is not applicable for categorical data. The variance of the test statistic is based on the hypergeometric distribution.

### Plasma cfDNA isolation and quantification of genome equivalents

At least 10 ml of whole blood was collected by blood draw using EDTA as anticoagulant. Plasma was separated within 5 h through two different centrifugation steps (the first at room temperature for 10 min at 1600×*g* and the second at 3000×*g* for the same time and temperature), obtaining up to 3 ml of plasma. Plasma was stored at −80 °C until cfDNA extraction. cfDNA was extracted from plasma using the QIAamp Circulating Nucleic Acid Kit (QIAGEN) according to the manufacturer’s instructions. An aliquot of 3 μl of cfDNA was used as template for each reaction. All samples were analyzed in duplicate. PCR reactions were performed using 10 μl final volume containing 5 μl LightCycler^®^480 SYBR Green I qPCR Master Mix, 2× (Roche) and LINE-1 (12,5 μmol) forward and reverse primers. DNA at known concentrations was also used to build the standard curve. Primer sequences are available on request.

### Droplet digital PCR

Measurement of FAM and HEX probes; gating was performed based on positive and negative controls, and mutant populations were identified. The ddPCR data were analyzed with QuantaSoft analysis software (Bio-Rad) to obtain fractional abundance of the mutant DNA alleles in the wild-type/normal background. The quantification of the target molecule was presented as number of total copies (mutant plus WT) per sample in each reaction. Fractional abundance is calculated as follows: F.A. % = (Nmut/(Nmut + Nwt)) × 100), where Nmut is number of mutant events and Nwt is number of WT events per reaction. Droplet digital PCR (﻿ddPCR) analysis of normal control DNA (human genomic DNA (Promega)) and no DNA template controls were always included. Leu13fs WT: forward sequence: 5′-CCGAGATGTCTCGCTC-3′; reverse sequence: 5′-GGAGGGTAGGAGAGACT-3′; probe sequence: 5′-CGCTACTCTCTCTTTCTGG-3′; fluorophore: HEX. Leu13fs del: forward sequence: 5′-CCGAGATGTCTCGCTC-3′; reverse sequence: 5′-GGAGGGTAGGAGAGACT-3′; probe sequence: 5′-CGCTACTCTCTTTCTGGC-3′; fluorophore: FAM.

### Generation of mouse melanoma cell line deficient for *B2M*

All lentiviruses were made using 293T cells transfected with lentiviral vectors, psPAX2 (Addgene 12260), and pMD2.G (Addgene 12259) at a 10:10:1 ratio, using TransIT-LT1 reagent (MIRUS, MIR 2300) according to the manufacturer’s instructions. Melanoma cell line derived from *BRAF* ^V600E^; *PTEN* ^−/−^ (BP) transgenic mice (a gift from the laboratory of David Fisher, MGH, Cancer Center) were initially transduced with the lentiCas9-EGFP virus (GPP Broad Institute, pXPR_BRD104) and GFP^high^-positive cells were sorted to generate BP^Cas9+GFP+^ cells. Cas9 activity was evaluated prior to transduction with each of the lentiGuide viruses using the Cas9 activity assay vector (GPP Broad Institute, pXPR_BRD011). Next, BP^Cas9+GFP+^ cells were transduced separately with a guide targeting *B2M* (5′-CACCG AGTATACTCACGCCACCCAC-3′, 5′-AAACGTGGGTGGCGTGAGTATACTC-3′) or a control guide against *LacZ* (5′-CACCGGTTCGCATTATCCGAACCAT-3′, 5′-AAACATGGTTCGGATAATGCGAACC-3′), cloned into a lentiGuide-Puro (pXPR_BRD003) or lentiGuide-Puro-Thy1.1 (modified pXPR-BRD003), respectively, with BsmBI compatible overhangs, using standard protocols. Cells were expanded and sorted after 5 days to establish the two cell lines clones: BP^Cas9+GFP+*B2M*−/−^ and BP^Cas9+GFP+Thy1.1+*B2M+/+*^. Flow cytometry was used to confirm the deletion of *B2M* and the expression of Thy1.1 in the two clones 5 days after sorting and only 0% B2M and 100% Thy1.1-positive cells were kept.

### Mice and tumor transplant and tissue collection

Female C57BL/6 mice 8–9 weeks of age were purchased form Jackson Laboratory and were housed at Massachusetts General Hospital under SPF conditions. Animal use followed protocols approved by the Massachusetts General Hospital Institutional Animal Care and Use Committee (IACUC). BP^Cas9+GFP+*B2M*−/−^ and BP^Cas9+GFP+Thy1.1+*B2M+/+*^ cells (1 × 10^6^) were mixed in a 1:1 ratio and injected intradermally into the right flank. For depletion assay, mice were treated with intraperitoneal injection of 200 µg anti-mouse NK1.1 (clone PK136, BioXCell, BP0036) on days 2 and 5 following tumor transplantation. On day +7, mice were sacrificed and tumors were dissected manually. Tumors were minced and digested with 2 mg/ml collagenase I (Sigma, C0130), 2 mg/mg hyaluronidase (Sigma, H3506) and 25 µg/ml Dnase (Promega) by incubating at 37 °C for 30 min with gentle mixing. Digests were then passed through 50 µm filter, counted and prepared for flow cytometry staining.

### Flow cytometry and antibodies

Monoclonal antibodies specific for CD16 and CD32 (Biolegend, 101302; 1:100) were used for blockade of Fc receptors before staining. The antibodies used for cell surface labeling were AF647 anti-mouse H-2Kb/H-2Db (Biolegend, 114612; 1:100), APC anti-mouse Thy1.1 (Biolegend, 202526; 1:100), and PE anti-mouse NK1.1 (Biolegend, 108707; 1:100). Intracellular staining of unlabeled B2M (Abcam, ab75853; 1:100) following staining with a secondary APC anti-rabbit IgG (R&D systems, F0111) was performed using a staining buffer set (eBioscience) according to the manufacturer’s instructions. Samples were analyzed by CytoFLEX flow cytometer (Beckman Coulter).

### In vivo competition assay

For the in vivo competition assay, BP^Cas9+GFP+*B2M*−/−^ and BP^Cas9+GFP+Thy1.1+*B2M+/+*^ cells were mixed in a 1:1 ratio and intradermally injected into the right flank of recipient mice. Tumors were collected on day 7 post transplantation and the ratio between each clone (GFP^+^Thy1.1^−^ or GFP^+^Thy1.1^+^) was determined based on flow cytometry analysis and was normalized based on the initial ratio measured on the day of tumor inoculation. The following formula was used to calculate the percentage change from baseline for the two clones: (1−(*B2M* ^−/−^ sample %/*B2M* ^−/−^ baseline %)/(B2M^+/+^ sample %/B2M^+/+^ baseline %)) × 100.

### Code availability

Information on Firehose can be found at http://www.broadinstitute.org/cancer/cga/Firehose. Software used in this study is available as standard pipelines on Firehose. For individual download, software used in this study can be found at http://www.broadinstitute.org/cancer/cga. ReCapSeg and AllelicCapseg can be downloaded from http://www.broadinstitute.org/cancer/cga/acsbeta. Mutect2 is found at https://software.broadinstitute.org/gatk/gatkdocs/org_broadinstitute_gatk_tools_walkers_cancer_m2_MuTect2.php. PhyloWGS v1.0-rc1 can be found at https://github.com/morrislab/phylowgs. NetMHCpan 2.4 can be found at http://www.cbs.dtu.dk/services/NetMHCpan-2.4/ ^42^.

### Data availability

The Van Allen data set referenced during the study is available in a public repository from dbGAP database (https://www.ncbi.nlm.nih.gov/gap), under accession code phs000452.v2.p1. The Hugo data set was provided to us by Roger S. Lo (rlo@mednet.ucla.edu) and is not available on a public repository. All sequencing data from this study have been deposited in dbGap database (https://www.ncbi.nlm.nih.gov/gap), under accession code phs001427.v1.p1.

## Electronic supplementary material

Supplementary Information

Peer Review File

Supplementary Files

Supplementary Data 1

Supplementary Data 2

Supplementary Data 3

Supplementary Data 4

Supplementary Data 5

## References

[1] Robert C (2015). Pembrolizumab versus ipilimumab in advanced melanoma. N. Engl. J. Med..

[2] Weber JS (2015). Nivolumab versus chemotherapy in patients with advanced melanoma who progressed after anti-CTLA-4 treatment (CheckMate 037): a randomised, controlled, open-label, phase 3 trial. Lancet Oncol..

[3] Herbst RS (2014). Predictive correlates of response to the anti-PD-L1 antibody MPDL3280A in cancer patients. Nature.

[4] Pardoll DM (2012). The blockade of immune checkpoints in cancer immunotherapy. Nat. Rev. Cancer.

[5] Zou W, Wolchok JD, Chen L (2016). PD-L1 (B7-H1) and PD-1 pathway blockade for cancer therapy: mechanisms, response biomarkers, and combinations. Sci. Transl. Med..

[6] Callahan MK, Postow MA, Wolchok JD (2016). Targeting T cell co-receptors for cancer therapy. Immunity.

[7] Hulpke S, Tampe R (2013). The MHC I loading complex: a multitasking machinery in adaptive immunity. Trends Biochem. Sci..

[8] Van Allen EM (2015). Genomic correlates of response to CTLA-4 blockade in metastatic melanoma. Science.

[9] Snyder A (2014). Genetic basis for clinical response to CTLA-4 blockade in melanoma. N. Engl. J. Med..

[10] Daud AI (2016). Programmed death-ligand 1 expression and response to the anti-programmed death 1 antibody pembrolizumab in melanoma. J. Clin. Oncol..

[11] Gao J (2016). Loss of IFN-gamma pathway genes in tumor cells as a mechanism of resistance to anti-CTLA-4 therapy. Cell.

[12] Zaretsky JM (2016). Mutations associated with acquired resistance to PD-1 blockade in melanoma. N. Engl. J. Med..

[13] Hodis E (2012). A landscape of driver mutations in melanoma. Cell.

[14] Hugo W (2016). Genomic and transcriptomic features of response to anti-PD-1 therapy in metastatic melanoma. Cell.

[15] Vesely MD, Schreiber RD (2013). Cancer immunoediting: antigens, mechanisms, and implications to cancer immunotherapy. Ann. N. Y. Acad. Sci..

[16] Tumeh PC (2014). PD-1 blockade induces responses by inhibiting adaptive immune resistance. Nature.

[17] Kloor M (2007). Beta2-microglobulin mutations in microsatellite unstable colorectal tumors. Int. J. Cancer.

[18] Bernal M, Ruiz-Cabello F, Concha A, Paschen A, Garrido F (2012). Implication of the beta2-microglobulin gene in the generation of tumor escape phenotypes. Cancer Immunol. Immunother..

[19] Porgador A, Mandelboim O, Restifo NP, Strominger JL (1997). Natural killer cell lines kill autologous beta2-microglobulin-deficient melanoma cells: implications for cancer immunotherapy. Proc. Natl Acad. Sci. USA.

[20] Raulet DH, Gasser S, Gowen BG, Deng W, Jung H (2013). Regulation of ligands for the NKG2D activating receptor. Annu. Rev. Immunol..

[21] Restifo NP (1996). Loss of functional beta 2-microglobulin in metastatic melanomas from five patients receiving immunotherapy. J. Natl Cancer Inst..

[22] Challa-Malladi M (2011). Combined genetic inactivation of beta2-microglobulin and CD58 reveals frequent escape from immune recognition in diffuse large B cell lymphoma. Cancer Cell.

[23] Berger MF (2012). Melanoma genome sequencing reveals frequent PREX2 mutations. Nature.

[24] Igney FH, Krammer PH (2002). Immune escape of tumors: apoptosis resistance and tumor counterattack. J. Leukoc. Biol..

[25] Maleno I (2011). Frequent loss of heterozygosity in the beta2-microglobulin region of chromosome 15 in primary human tumors. Immunogenetics.

[26] Wagle N (2014). Response and acquired resistance to everolimus in anaplastic thyroid cancer. N. Engl. J. Med..

[27] Fisher S (2011). A scalable, fully automated process for construction of sequence-ready human exome targeted capture libraries. Genome Biol..

[28] Dobin A (2013). STAR: ultrafast universal RNA-seq aligner. Bioinformatics.

[29] Li B, Dewey CN (2011). RSEM: accurate transcript quantification from RNA-Seq data with or without a reference genome. BMC Bioinformatics.

[30] Chapman MA (2011). Initial genome sequencing and analysis of multiple myeloma. Nature.

[31] Saunders CT (2012). Strelka: accurate somatic small-variant calling from sequenced tumor-normal sample pairs. Bioinformatics.

[32] Lai Z (2016). VarDict: a novel and versatile variant caller for next-generation sequencing in cancer research. Nucleic Acids Res..

[33] Cibulskis K (2013). Sensitive detection of somatic point mutations in impure and heterogeneous cancer samples. Nat. Biotechnol..

[34] Ramos AH (2015). Oncotator: cancer variant annotation tool. Hum. Mutat..

[35] Carter SL (2012). Absolute quantification of somatic DNA alterations in human cancer. Nat. Biotechnol..

[36] Deshwar AG (2015). PhyloWGS: reconstructing subclonal composition and evolution from whole-genome sequencing of tumors. Genome Biol..

[37] Shukla SA (2015). Comprehensive analysis of cancer-associated somatic mutations in class I HLA genes. Nat. Biotechnol..

[38] Hoof I (2009). NetMHCpan, a method for MHC class I binding prediction beyond humans. Immunogenetics.

[39] Cerami E (2012). The cBio cancer genomics portal: an open platform for exploring multidimensional cancer genomics data. Cancer Discov..

[40] Gao J (2013). Integrative analysis of complex cancer genomics and clinical profiles using the cBioPortal. Sci. Signal..

[41] Schindelin J (2012). Fiji: an open-source platform for biological-image analysis. Nat. Methods.

[42] Amit M (2014). Local exact pattern matching for non-fixed RNA structures. IEEE/ACM Trans. Comput. Biol. Bioinform..

