# Supplementary material for: Resistance to checkpoint blockade therapy through inactivation of antigen presentation
Source: Nat Commun. 2017 Oct 26;8:1136. doi: 10.1038/s41467-017-01062-w (PMC5656607; doi:10.1038/s41467-017-01062-w)
Supplement: Supplementary file 1 — Supplementary Information [file 41467_2017_1062_MOESM1_ESM.pdf]

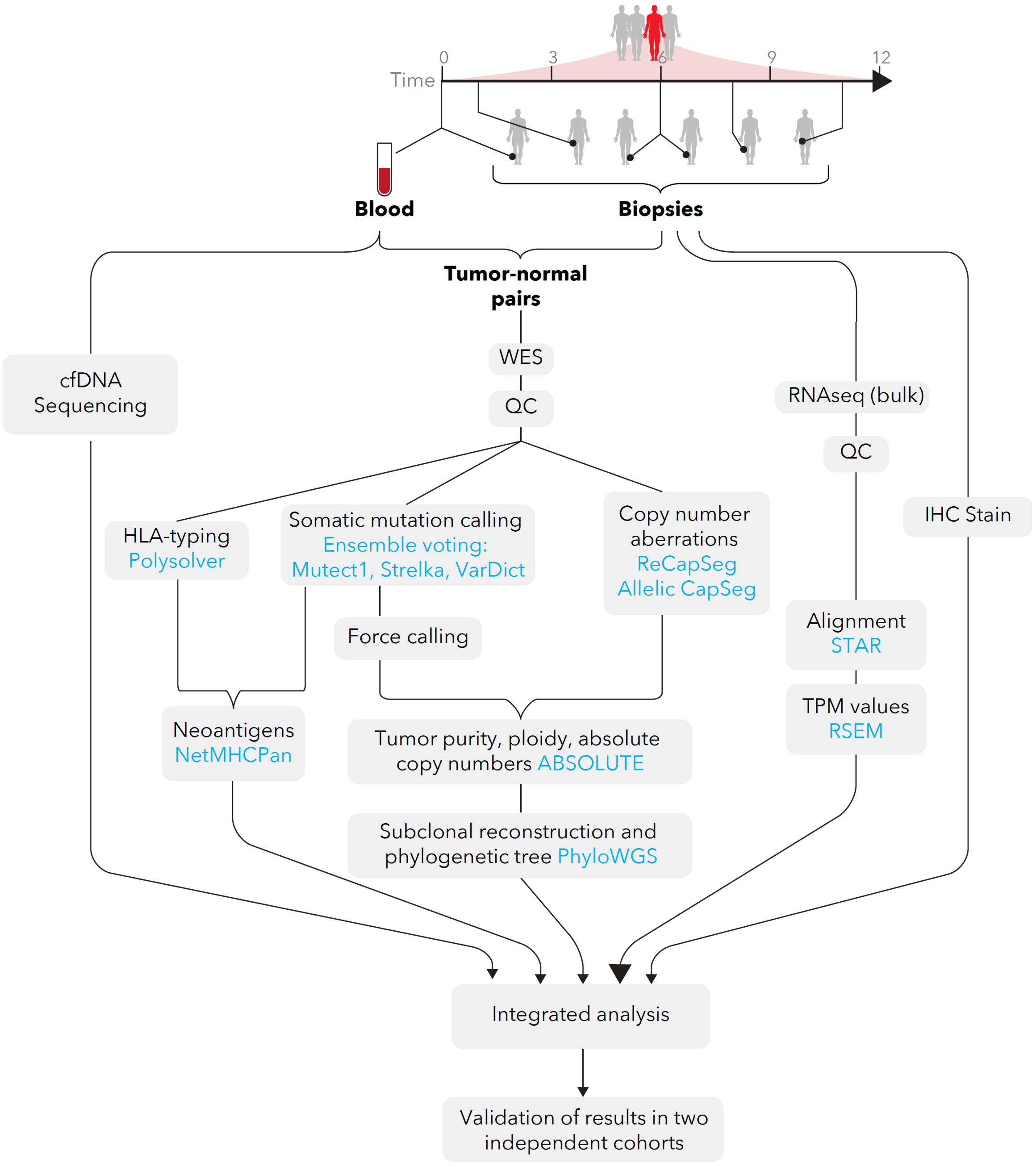

Supplementary Figure 1 | Overview of the experimental and computational workflow for this study. Major analysis steps are shown in gray boxes. Names of software used for each step are shown in light blue.

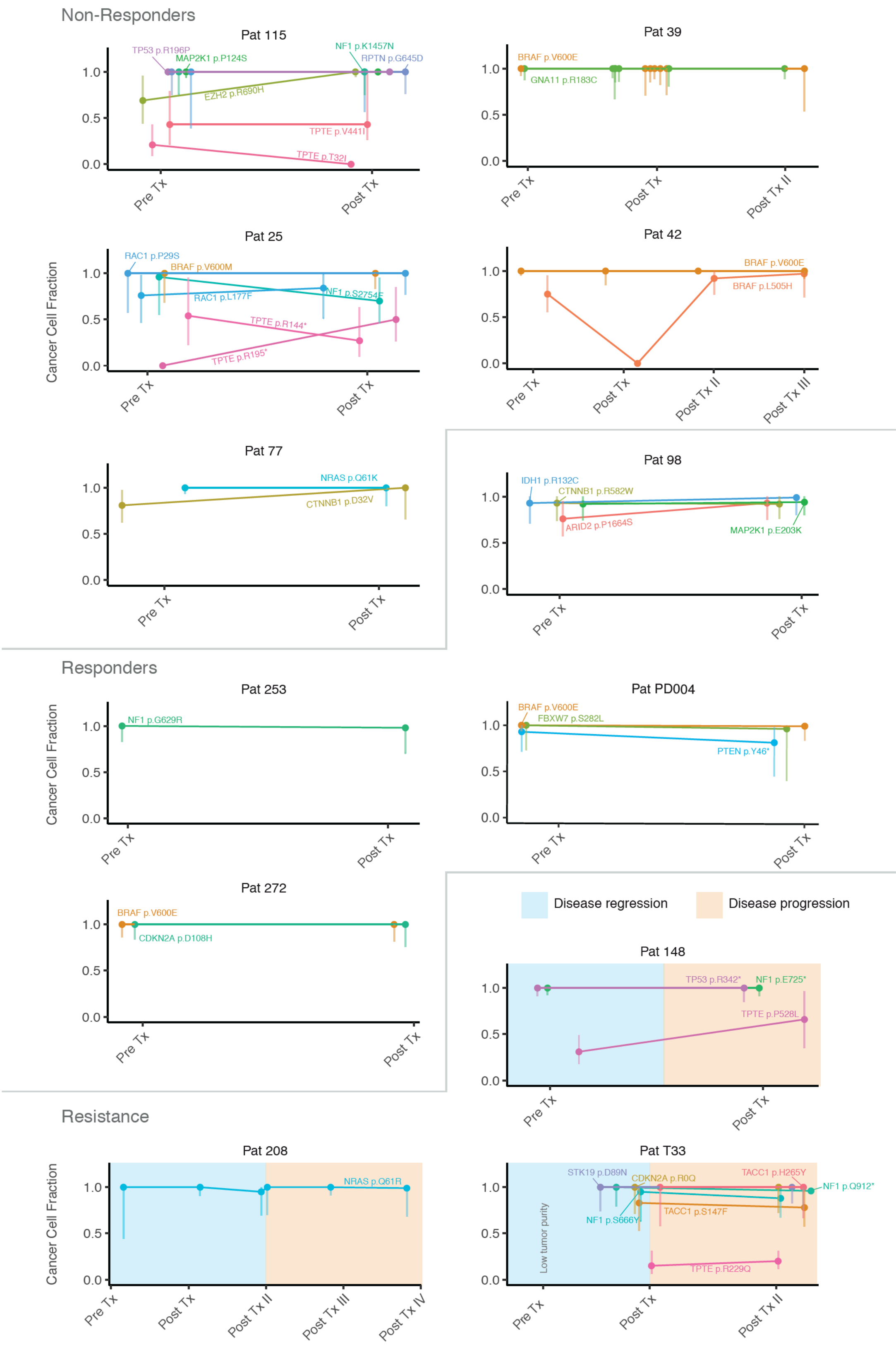
Non-Responders
Pat 115
TP53 p.R196P
MAP2K1 p.P124S
NF1 p.K1457N
RPTN p.G645D
EZH2 p.R690H
TPTE p.V441I
TPTE p.T32I
Pre Tx
Post Tx
Pat 39
BRAF p.V600E
GNA11 p.R183C
Pre Tx
Post Tx
Post Tx II
Pat 25
RAC1 p.P29S
BRAF p.V600M
RAC1 p.L177F
NF1 p.S2754F
TPTE p.R144*
TPTE p.R195*
Pre Tx
Post Tx
Pat 42
BRAF p.V600E
BRAF p.L505H
Pre Tx
Post Tx
Post Tx II
Post Tx III
Pat 77
NRAS p.Q61K
CTNNB1 p.D32V
Pre Tx
Post Tx
Pat 98
IDH1 p.R132C
CTNNB1 p.R582W
ARID2 p.P1664S
MAP2K1 p.E203K
Pre Tx
Post Tx
Responders
Pat 253
NF1 p.G629R
Pre Tx
Post Tx
Pat PD004
BRAF p.V600E
FBXW7 p.S282L
PTEN p.Y46*
Pre Tx
Post Tx
Pat 272
BRAF p.V600E
CDKN2A p.D108H
Pre Tx
Post Tx
Disease regression
Disease progression
Pat 148
TP53 p.R342*
NF1 p.E725*
TPTE p.P528L
Pre Tx
Post Tx
Resistance
Pat 208
NRAS p.Q61R
Pre Tx
Post Tx
Post Tx II
Post Tx III
Post Tx IV
Pat T33
STK19 p.D89N
CDKN2A p.R0Q
TACC1 p.H265Y
NF1 p.Q912*
NF1 p.S666Y
TACC1 p.S147F
TPTE p.R229Q
Low tumor purity
Pre Tx
Post Tx
Post Tx II
Cancer Cell Fraction
1.0
0.5
0.0

Supplementary Figure 2 | The cancer cell fraction (CCFs) of most known melanoma drivers did not change significantly over time in tumor biopsies. Panels display CCF values for known melanoma drivers in 12 patients out of 17. No known melanoma related drivers were detected in Pat155, Pat62, Pat51, Pat99 and Pat131.

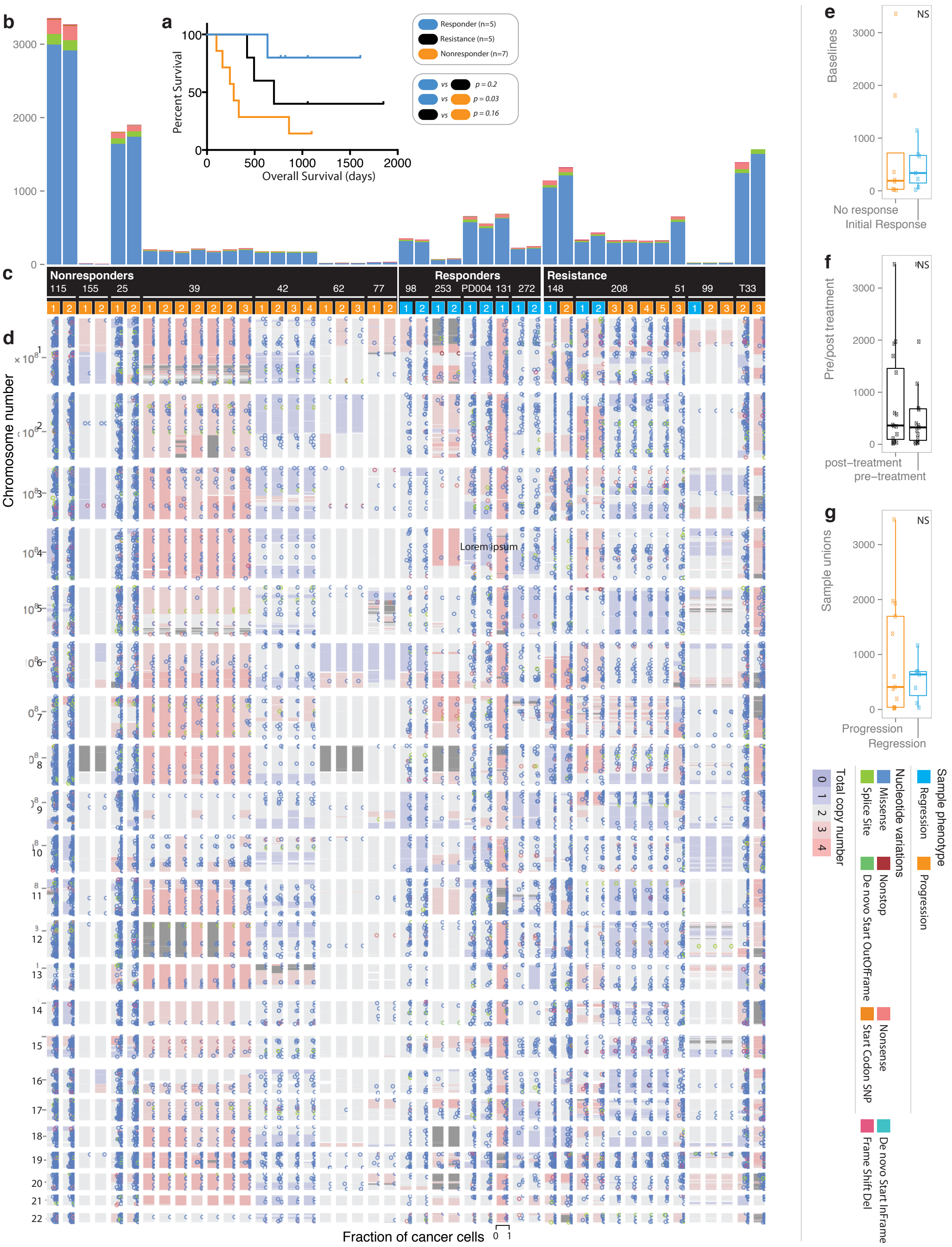

a
Percent Survival
Overall Survival (days)
Responder (n=5)
Resistance (n=5)
Nonresponder (n=7)
vs p = 0.2
vs p = 0.03
vs p = 0.16
b
c
Nonresponders
Responders
Resistance
d
Chromosome number
Lorem ipsum
Fraction of cancer cells
e
Baselines
No response
Initial Response
NS
f
Pre/post treatment
post−treatment
pre−treatment
NS
g
Sample unions
Progression
Regression
NS
Sample phenotype
Regression
Progression
Nucleotide variations
Missense
Nonstop
Nonsense
De novo Start InFrame
Splice Site
De novo Start OutOfFrame
Start Codon SNP
Frame Shift Del
Total copy number

Supplementary Figure 3 | Summary of all nucleotide and copy number variants found in our dataset. (**a**) Overall survival of patients treated with CPB within our cohort whose tumors responded (n=5), did not respond (n=10) or relapsed after an initial response (n=5). Log-rank p-value is shown (**b**) Stacked bar plot summarizing the total number of nucleotide variants per patient. Different colors represent different types of nucleotie variants. (**c**) Patient information. First row (white text on black background) indicate patient classification as either nonresponder, responder, or resistance. Second row (white text on black background) are patient IDs. Third row (white text on orange or blue backgrounds) indicate time points at which biopsies were taken from each patient. Orange, biopsy was taken prior to or during progression; blue, biopsy was taken prior to or during regression. (**d**) Summary of nucleotide and copy number variations. Each rectangular region indicates a chromosome. Amplifications and deletions are shown as red and blue, respectively. The chromosomal location of each nucleotide variant is shown on the verticle axis. The cancer cell fraction of the variant is shown on the horizontal axis, with left indicating frequency of 0 and right indicating 1. (**e**) Boxplot of mutation load in baseline samples from patients who did (blue) or did not (orange) show initial response to CPB. (**f**) Boxplot of mutation load in samples prior to or after treatment. If multiple samples were available post-treatment for a single patient, the mutation load was calculated as the union of the sets of mutations found in post-treatment samples. (**g**) Boxplot of mutation load in samples taken during disease progression (orange) or regression (blue). If multiple samples were available from a single patient during either progression or regression, the mutation load was calculated as the union of the sets of mutations found in each sample.

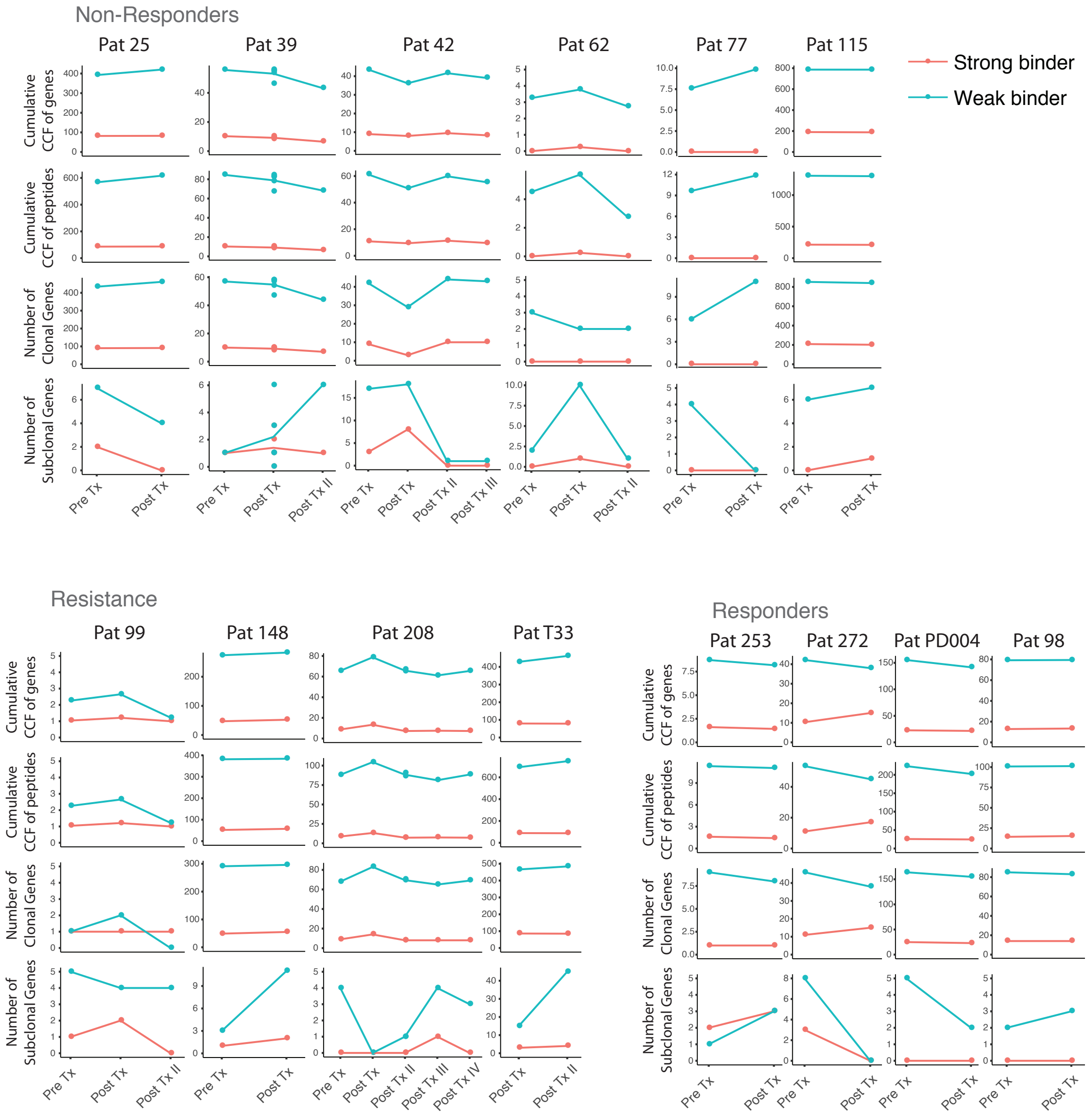
Non-Responders
Pat 25
Pat 39
Pat 42
Pat 62
Pat 77
Pat 115
Strong binder
Weak binder
Cumulative CCF of genes
Cumulative CCF of peptides
Number of Clonal Genes
Number of Subclonal Genes
Pre Tx
Post Tx
Post Tx II
Post Tx III
Resistance
Pat 99
Pat 148
Pat 208
Pat T33
Post Tx IV
Responders
Pat 253
Pat 272
Pat PD004
Pat 98

Supplementary Figure 4 | Neoantigen load in patients treated with CPB. Each plot displays the neoantigen load over time per patient. For each patient, in the top row, CCF values for genes that contributed at least one neoantigen were summed. In the second row, CCF values for each predicted antigenic peptide was summed, such that genes contributing multiple neoantigens would be represented multiple times. In the third row, the number of clonal genes that contributed at least one neoantigen was calculated. In the last row, the number of subclonal genes that contributed at least one neoantigen was calculated. Red lines indicate strong binders, blue lines indicate weak binders.

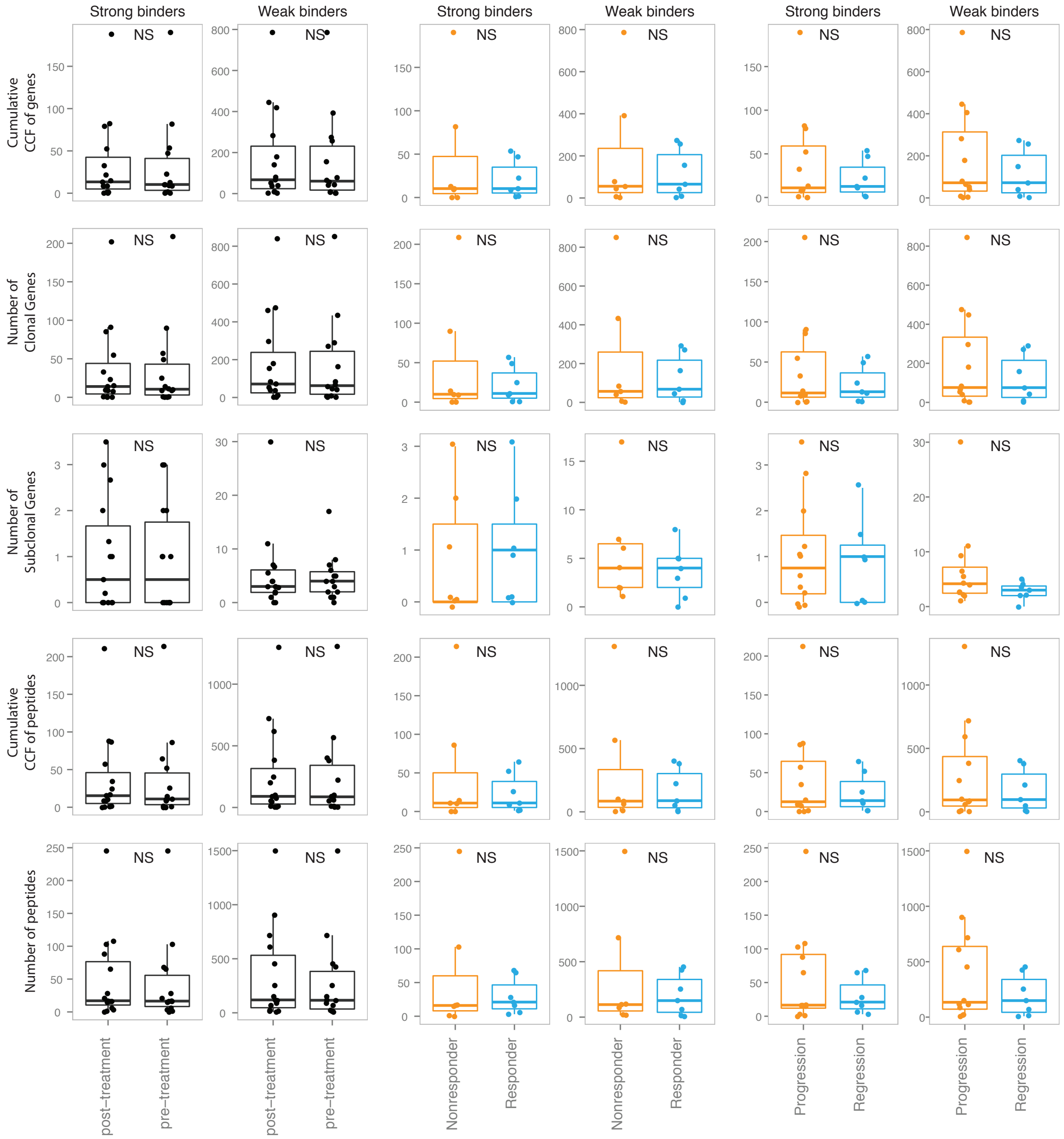

Strong binders
Weak binders
Strong binders
Weak binders
Strong binders
Weak binders
Cumulative CCF of genes
Number of Clonal Genes
Number of Subclonal Genes
Cumulative CCF of peptides
Number of peptides
NS
post−treatment
pre−treatment
Nonresponder
Responder
Progression
Regression

Supplementary Figure 5 | Summary of neoantigen loads in our dataset. Boxplots of predicted strong binders and weak binders are shown for various sample groups (columns) and neoantigen load metrics (rows). Rows show, from top to bottom: the sum of CCF values for genes that contributed at least one neoantigen (first row); sum of CCF values for each predicted antigenic peptide, such that genes contributing multiple neoantigens would be represented multiple times (second row); the number of clonal genes that contributed at least one neoantigen (third row); and the number of subclonal genes that contributed at least one neoantigen (fourth row). Columns show, from left to right: neoantigen load between pre and post-treatment samples (first and second columns); baseline samples from responders and nonresponders (third and fourth columns); and samples taken during disease progression and regression (fifth and sixth columns). If multiple samples from a single patient were available for any grouping, the neoantigen load was calculated from the union of the sets of mutations found in each sample.

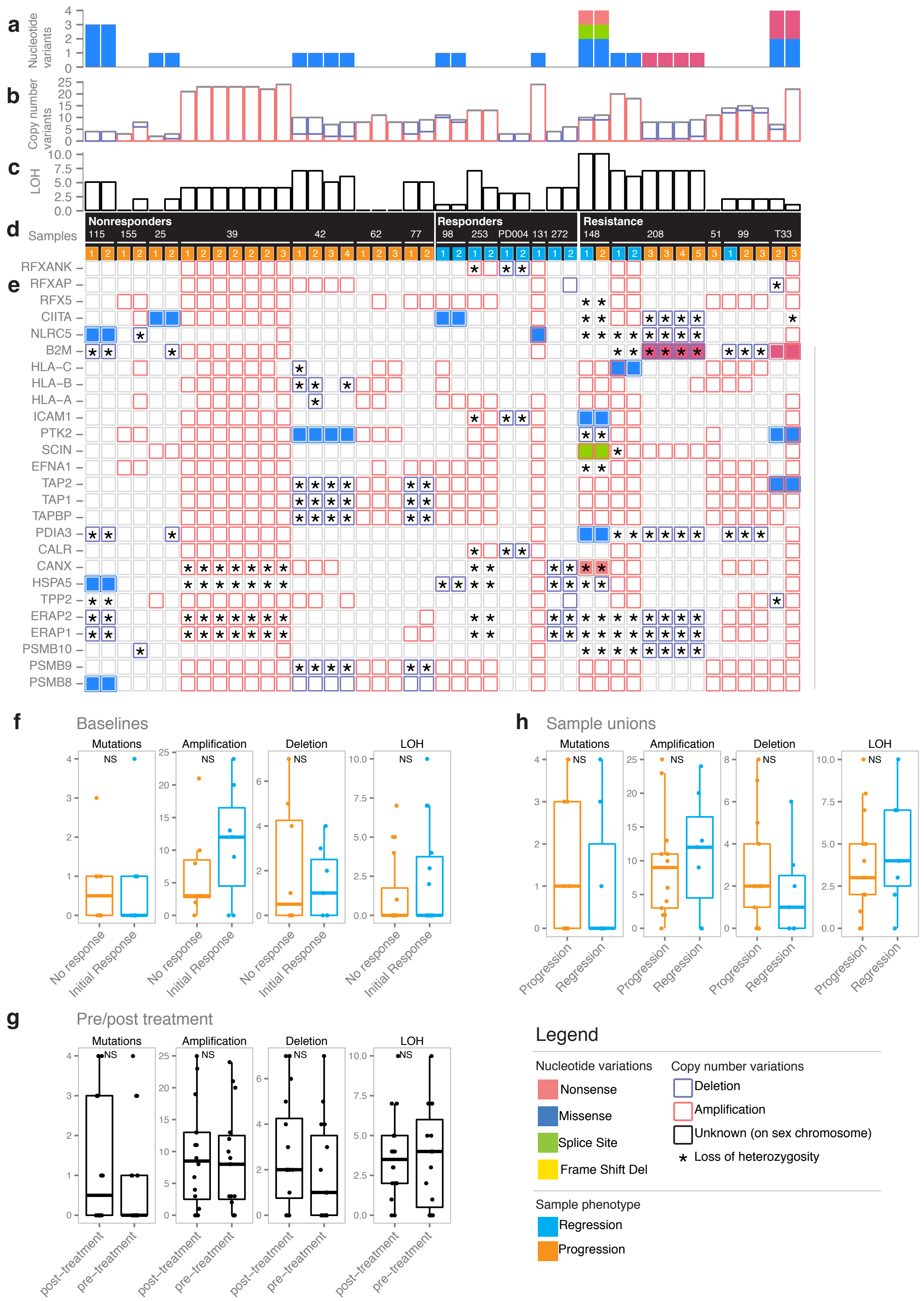

a
Nucleotide variants
b
Copy number variants
c
LOH
d
Samples
Nonresponders
Responders
Resistance
e
RFXANK
RFXAP
RFX5
CIITA
NLRC5
B2M
HLA–C
HLA–B
HLA–A
ICAM1
PTK2
SCIN
EFNA1
TAP2
TAP1
TAPBP
PDIA3
CALR
CANX
HSPA5
TPP2
ERAP2
ERAP1
PSMB10
PSMB9
PSMB8
f
Baselines
Mutations
Amplification
Deletion
LOH
No response
Initial Response
h
Sample unions
Progression
Regression
g
Pre/post treatment
post–treatment
pre–treatment
Legend
Nucleotide variations
Nonsense
Missense
Splice Site
Frame Shift Del
Copy number variations
Deletion
Amplification
Unknown (on sex chromosome)
Loss of heterozygosity
Sample phenotype
Regression
Progression

Supplementary Figure 6 | Summary of nucleotide and copy number variants found in the HLA presentation pathway. (**a**) Stacked bar plot summarizing the total number of nucleotide variants with cancer cell fraction ≥ 0.5 found in the HLA pathway sets per patient. Colors indicate different types of nucleotide variants. (**b**) Stacked bar plot summarizing the total number of copy number variants found in the HLA pathway per patient. Red line, amplification; blue line, deletion. (**c**) Stacked bar plot summarizing the total number of LOH found in the HLA pathway per patient. (**d**) Patient information. First row (white text on black background) indicate patient classification as either nonresponder, responder, or resistance. Second row (white text on black background) are patient IDs. Third row (white text on orange or blue backgrounds) indicate time points at which biopsies were taken from each patient. Orange, biopsy was taken prior to or during progression; blue, biopsy was taken prior to or during regression. (**e**) Summary of nucleotide and copy number variants in the HLA pathway per sample. Nucleotide variants are displayed as fill colors. Only nucleotide variants with cancer cell fraction ≥ 0.5 are shown. Copy number variants are displayed as lines. Colors are defined as in **a** and **b**. (**f**) Boxplot of nucleotide and copy number variants in baseline samples from patients who did (blue) or did not (orange) show initial response to CPB. (**g**) Boxplot of nucleotide and copy number variants in samples prior to or after treatment. If multiple samples were available post-treatment for a single patient, the mutation load was calculated as the union of the sets of mutations found in post-treatment samples. (**h**) Boxplot of nucleotide and copy number variants in samples taken during disease progression (orange) or regression (blue). If multiple samples were available from a single patient during either progression or regression, the mutation load was calculated as the union of the sets of mutations found in each sample.

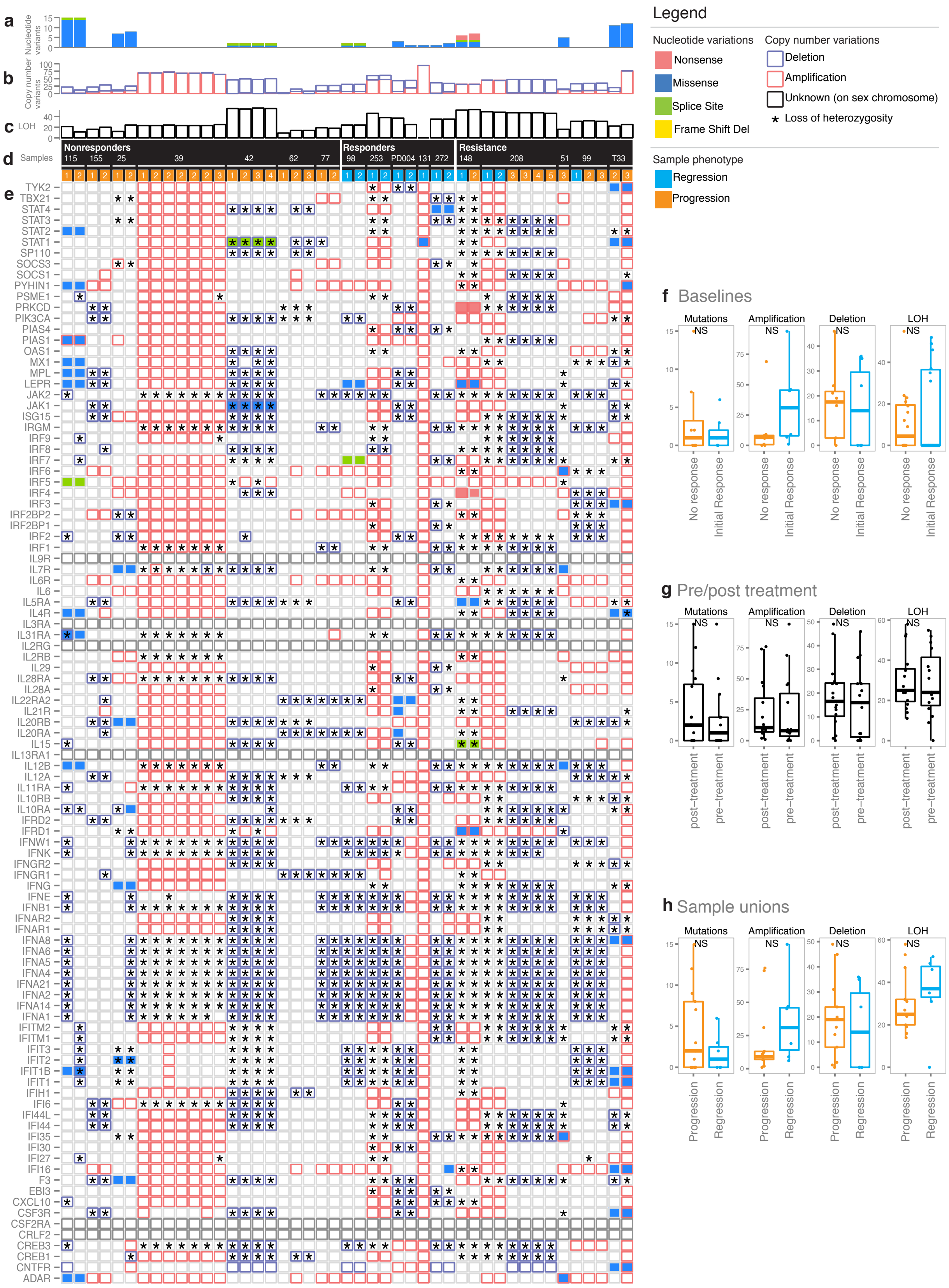

a
Nucleotide variants
b
Copy number variants
c
LOH
d
Samples
Nonresponders
Responders
Resistance
115
155
25
39
42
62
77
98
253
PD004
131
272
148
208
51
99
T33
e
TYK2
TBX21
STAT4
STAT3
STAT2
STAT1
SP110
SOCS3
SOCS1
PYHIN1
PSME1
PRKCD
PIK3CA
PIAS4
PIAS1
OAS1
MX1
MPL
LEPR
JAK2
JAK1
ISG15
IRGM
IRF9
IRF8
IRF7
IRF6
IRF5
IRF4
IRF3
IRF2BP2
IRF2BP1
IRF2
IRF1
IL9R
IL7R
IL6R
IL6
IL5RA
IL4R
IL3RA
IL31RA
IL2RG
IL2RB
IL29
IL28RA
IL28A
IL22RA2
IL21R
IL20RB
IL20RA
IL15
IL13RA1
IL12B
IL12A
IL11RA
IL10RB
IL10RA
IFRD2
IFRD1
IFNW1
IFNK
IFNGR2
IFNGR1
IFNG
IFNE
IFNB1
IFNAR2
IFNAR1
IFNA8
IFNA6
IFNA5
IFNA4
IFNA21
IFNA2
IFNA14
IFNA1
IFITM2
IFITM1
IFIT3
IFIT2
IFIT1B
IFIT1
IFIH1
IFI6
IFI44L
IFI44
IFI35
IFI30
IFI27
IFI16
F3
EBI3
CXCL10
CSF3R
CSF2RA
CRLF2
CREB3
CREB1
CNTFR
ADAR
Legend
Nucleotide variations
Nonsense
Missense
Splice Site
Frame Shift Del
Copy number variations
Deletion
Amplification
Unknown (on sex chromosome)
Loss of heterozygosity
Sample phenotype
Regression
Progression
f Baselines
Mutations
Amplification
Deletion
LOH
NS
No response
Initial Response
g Pre/post treatment
post−treatment
pre−treatment
h Sample unions
Progression
Regression

Supplementary Figure 7 | Summary of nucleotide and copy number variants found in the interferon-gamma pathway. (**a**) Stacked bar plot summarizing the total number of nucleotide variants with cancer cell fraction ≥ 0.5 found in the interferon-gamma pathway sets per patient. Colors indicate different types of nucleotide variants. (**b**) Stacked bar plot summarizing the total number of copy number variants found in the interferon-gamma pathway per patient. Red line, amplification; blue line, deletion. (**c**) Stacked bar plot summarizing the total number of LOH found in the interferon-gamma pathway per patient. (**d**) Patient information. First row (white text on black background) indicate patient classification as either nonresponder, responder, or resistance. Second row (white text on black background) are patient IDs. Third row (white text on orange or blue backgrounds) indicate time points at which biopsies were taken from each patient. Orange, biopsy was taken prior to or during progression; blue, biopsy was taken prior to or during regression. (**e**) Summary of nucleotide and copy number variants in the interferon-gamma pathway per sample. Nucleotide variants are displayed as fill colors. Only nucleotide variants with cancer cell fraction ≥ 0.5 are shown. Copy number variants are displayed as lines. Colors are defined as in **a** and **b**. (**f**) Boxplot of nucleotide and copy number variants in baseline samples from patients who did (blue) or did not (orange) show initial response to CPB. (**g**) Boxplot of nucleotide and copy number variants in samples prior to or after treatment. If multiple samples were available post-treatment for a single patient, the mutation load was calculated as the union of the sets of mutations found in post-treatment samples. (**h**) Boxplot of nucleotide and copy number variants in samples taken during disease progression (orange) or regression (blue). If multiple samples were available from a single patient during either progression or regression, the mutation load was calculated as the union of the sets of mutations found in each sample.

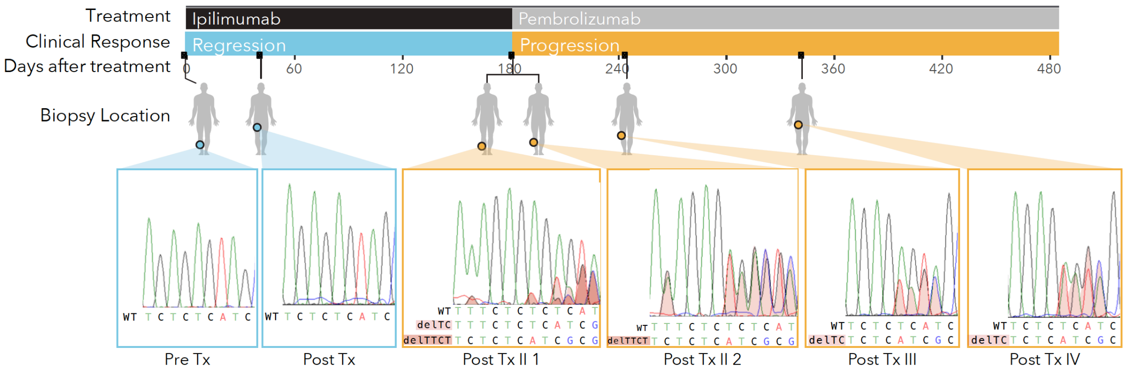

Supplementary Figure 8 | Identification of *B2M* mutations by Sanger sequencing. Sanger sequencing of the region surrounding the identified *B2M* mutations was performed for six biopsies taken from Pat208. Regression biopsies are outlined in blue, and progression biopsies are outlined in orange. Regions where reads support mutations are colored in light red. Ipilimumab- anti-CTLA4, Pembrolizumab- anti-PD1.

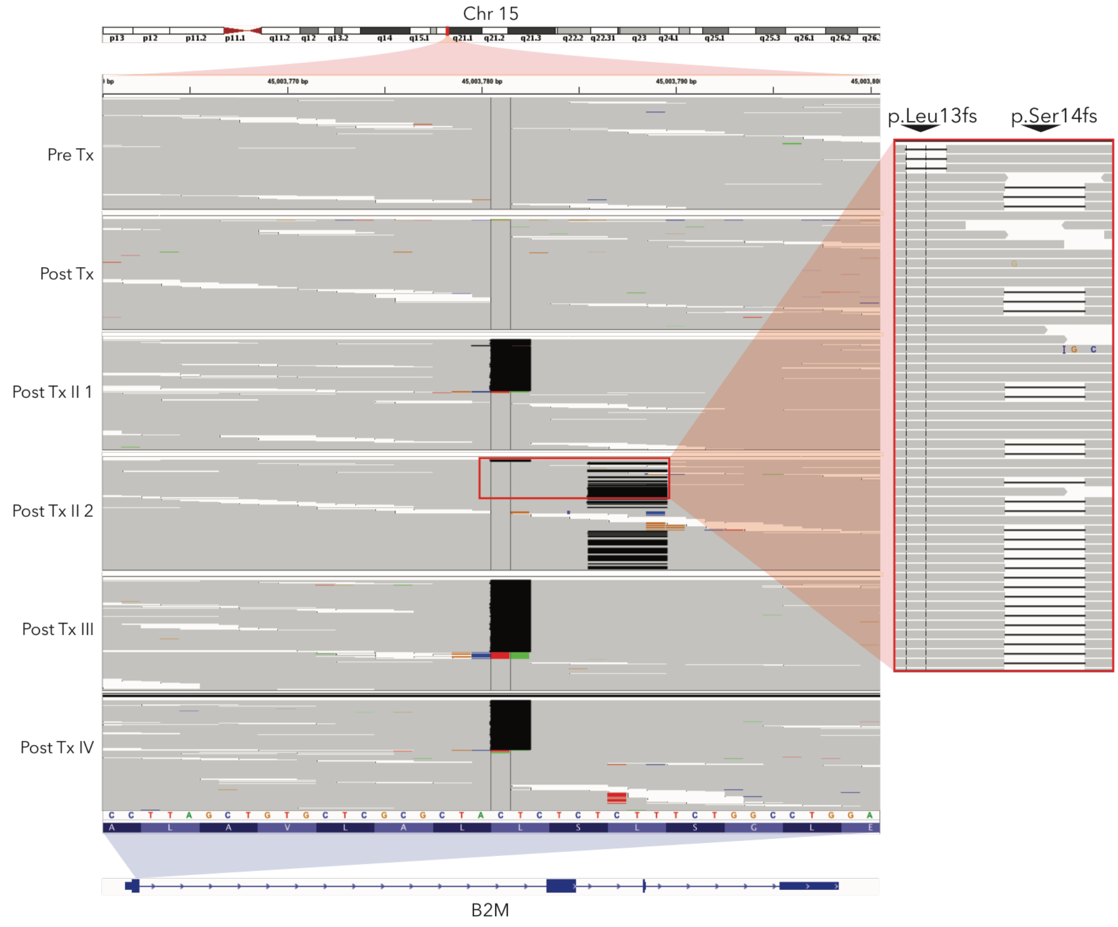

Supplementary Figure 9 | IGV plot of Pat208. BAM files containing sequencing data from Pat208 biopsies were viewed in the Integrated Genome Browser (IGV). The region of chr15:45,003,761-45,003,800, containing the two frameshift mutations in *B2M,* is shown. Black bars indicate reads supporting deletions. Reads are sorted by the base in between the black vertical lines at the center of the screenshot, thus all reads supporting deletions are shown at top. Biopsy names are shown on the left. The inset shows an expanded view of reads in Post Tx II 2 biopsy, highlighting the coexistence of the reads in both p.Leu13fs and p.Ser14fs. No reads support both mutations.

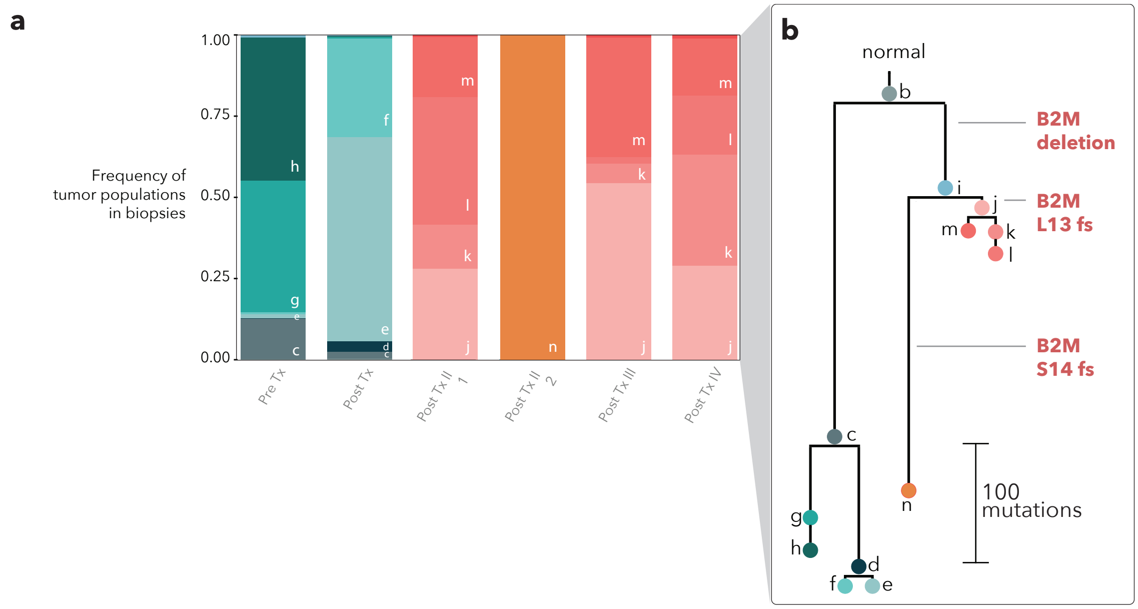

Supplementary Figure 10 | Inferred evolutionary history of tumor populations found in Pat208. (**a**) The subclonal composition of each biopsy from Pat208 as inferred by phyloWGS (described in **Methods**). Each color indicates a separate population in the tumor. (**b**) The phylogenetic relationships between populations shown in **a**.

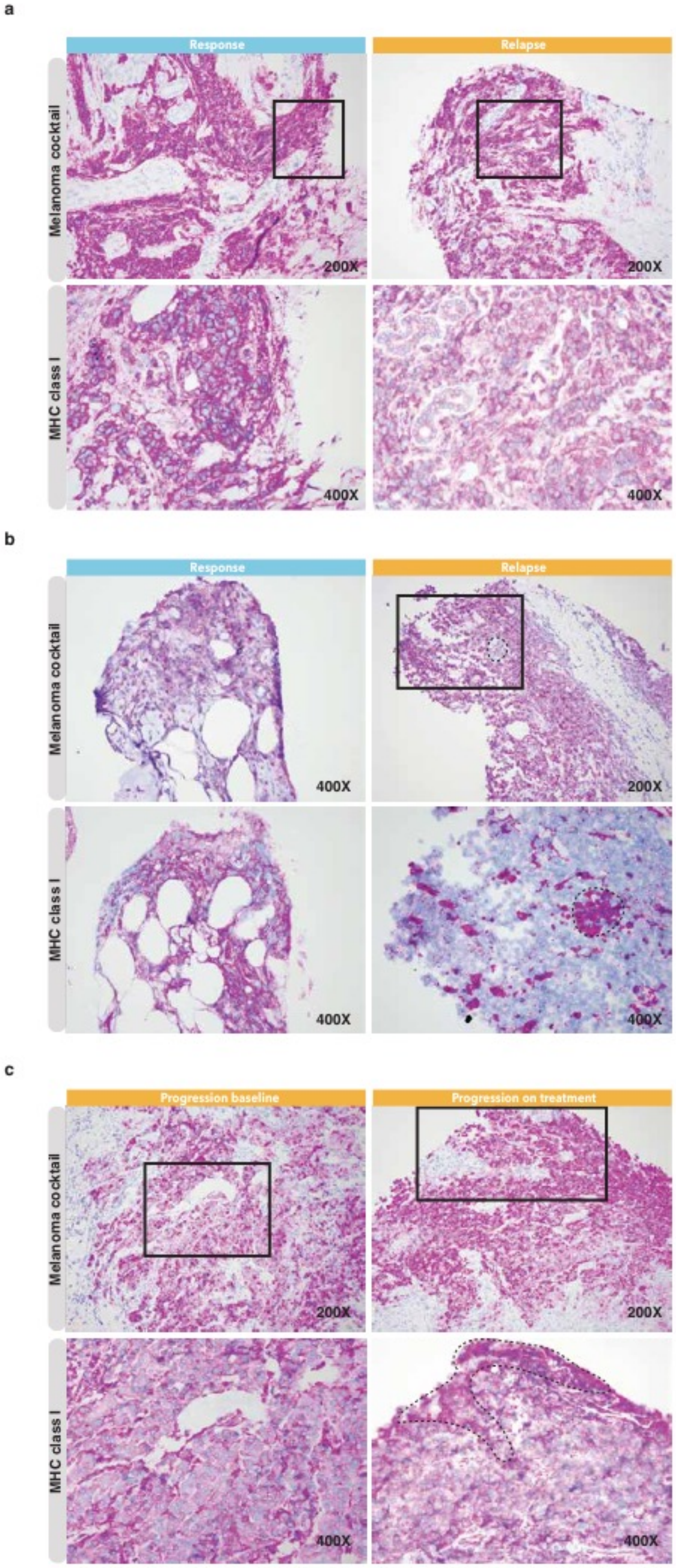
a
Response
Relapse
Melanoma cocktail
MHC class I
200X
200X
400X
400X
b
Response
Relapse
Melanoma cocktail
MHC class I
400X
200X
400X
400X
c
Progression baseline
Progression on treatment
Melanoma cocktail
MHC class I
200X
200X
400X
400X

Supplementary Figure 11 | Immunohistochemical staining of MHC class I in patients with *B2M* aberration. (**a-c**) Samples during disease regression or progression form Pat208 (**a**), Pat99 (**b**) and Pat25 (**c**) were stained with an antibody cocktail for melanoma cells (melanoma cocktail) using anti-melanosome (HMB45), anti-MART-1/melan A and anti-Tyrosinase, to discern melanoma cells from normal cells; or with a pan-MHC class I antibody. Panels show lack of MHC class I localization to the outer cells membrane of melanoma positive cells only during disease progression when B2M expression was lost (as seen in **Figures 1d** and **Figure 3 b-c**). Boxes areas in the upper panels (melanoma cocktail, original magnification X200) are shown at higher magnification (X400) in the lower panels (MHC class I). Dashed lines show areas that do not contain tumor cells.

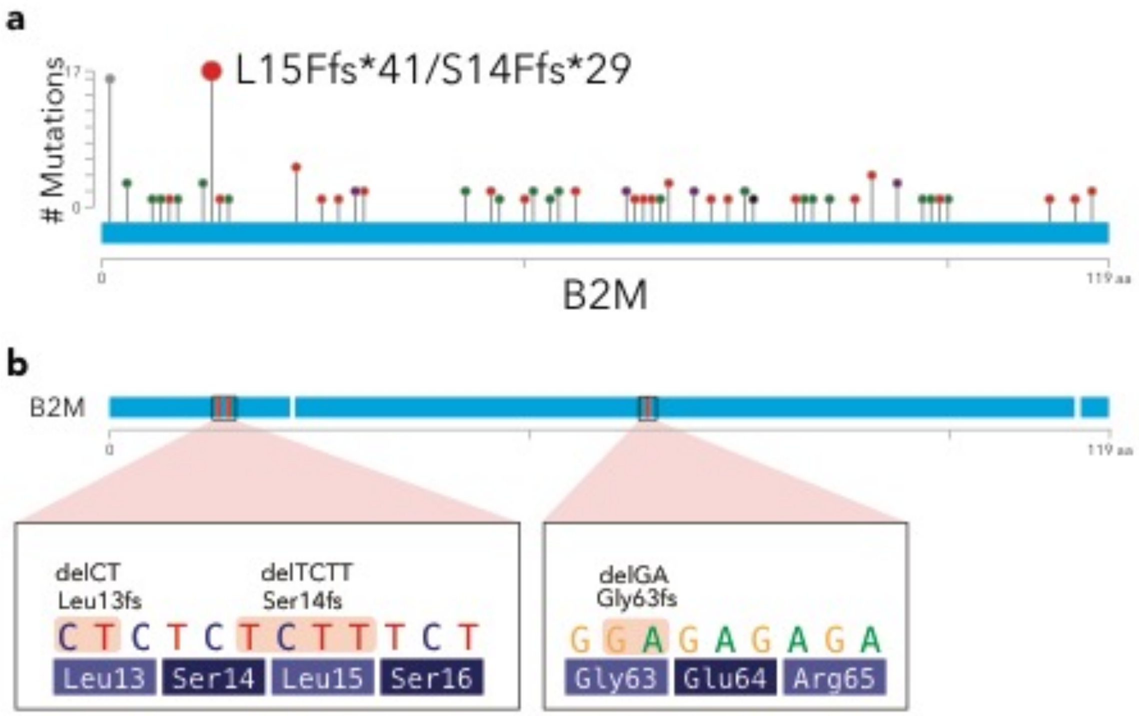

Supplementary Figure 12 | *B2M* mutational hotspot seen in TCGA data lie within 4X dinucleotide repeats. Long blue rectangles indicate exons of *B2M*. (**a**) The number of *B2M* mutations found in TCGA along *B2M* exons. A hotspot at p.Leu15 and p.Leu14 is labeled. (**b**) *B2M* mutations found in Pat208 and PatT33. Insets display the DNA sequence and amino acids at boxed locations, showing 4x dinucleotide repeats.

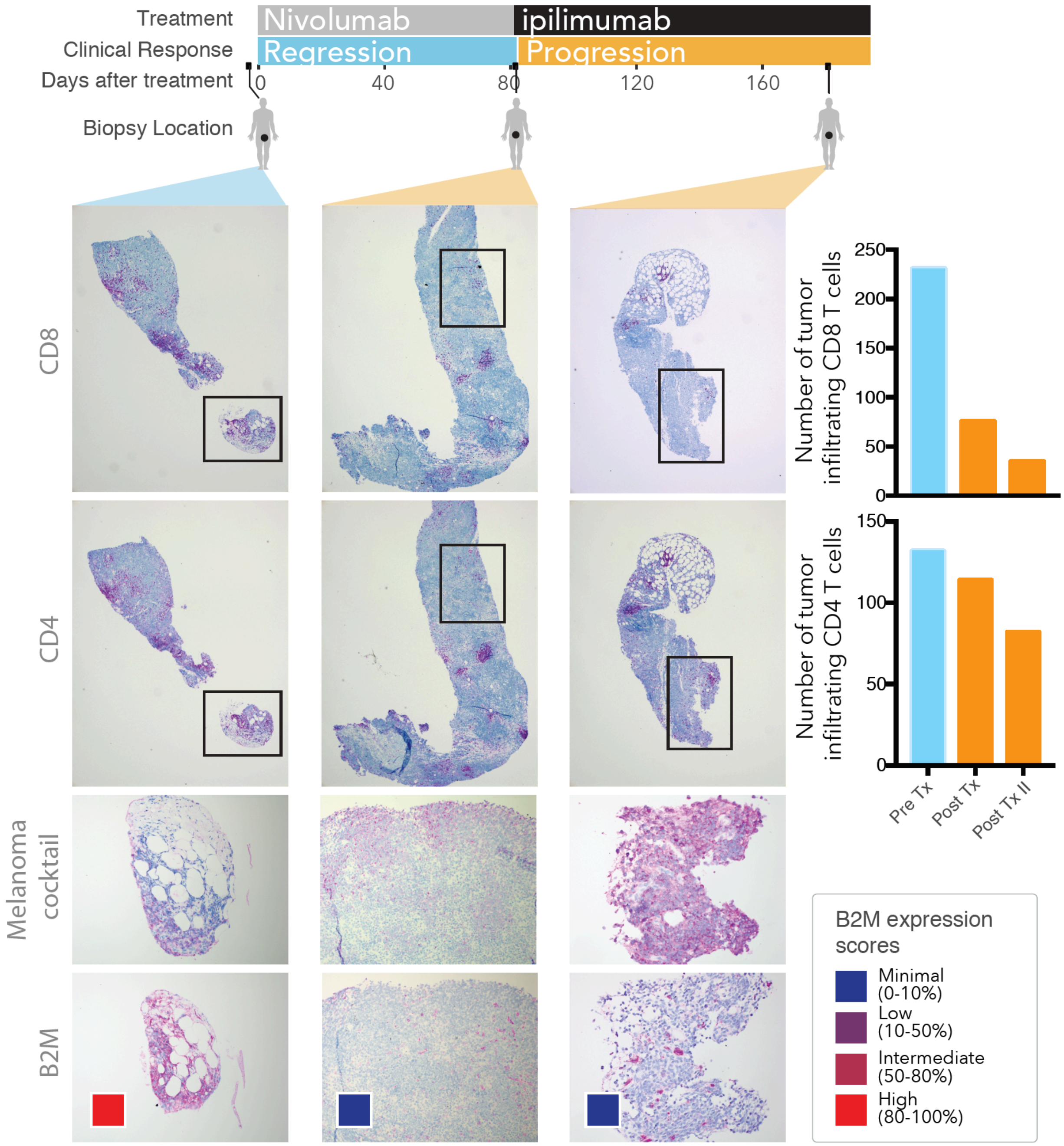

Treatment
Nivolumab
ipilimumab
Clinical Response
Regression
Progression
Days after treatment
0
40
80
120
160
Biopsy Location
CD8
CD4
Melanoma cocktail
B2M
Number of tumor infiltrating CD8 T cells
250
200
150
100
50
0
Number of tumor infiltrating CD4 T cells
150
100
50
0
Pre Tx
Post Tx
Post Tx II
B2M expression scores
Minimal (0-10%)
Low (10-50%)
Intermediate (50-80%)
High (80-100%)

Supplementary Figure 13 | Immunohistochemical staining of CD4 and CD8 in Pat99. Samples for each time point from Pat99 were stained with specific antibodies against CD8 or CD4 and were quantified using the cell counter function in Fiji (described in **Methods**). Boxes areas in the upper panels (CD4 and CD8, original magnification X40) are shown at higher magnification (X100) in the lower panels (melanoma coacktial and B2M). Colored boxes indicate B2M expression scores: B2M scoring was estimated by using 4 different levels of expression in the tumor fraction: Minimal- 0-10%; Low- 10-50%; Intermediate- 50-80%; and High- 80-100%, B2M expression in the tumor fraction. A timeline of treatment, clinical response, and biopsy locations is shown at the top.

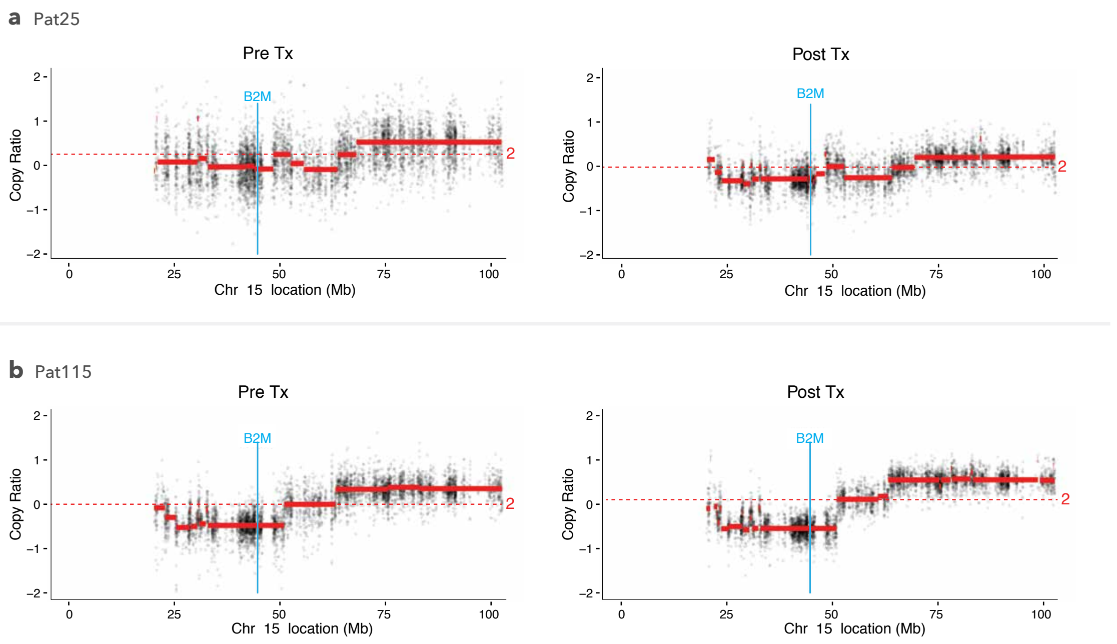

Supplementary Figure 14 | Total Copy Number from Biopsies Taken From non-responders shows a deletion overlapping the *B2M* locus. (**a-b**) Results from ReCapSeg of pre-CPB and post-CPB biopsies from two non-responders, Pat25 (**a**) and Pat115 (**b**), are shown. The horizontal axis indicates position on chromosome 15. The vertical axis indicates the total copy number ratio of targets. Each dot indicates a target region used to calculate copy ratio information. Solid red horizontal lines are segments inferred by the ReCapSeg algorithm. Dashed red lines indicate copy ratio corresponding to a total copy number of two, as inferred by ABSOLUTE.

a Pat99

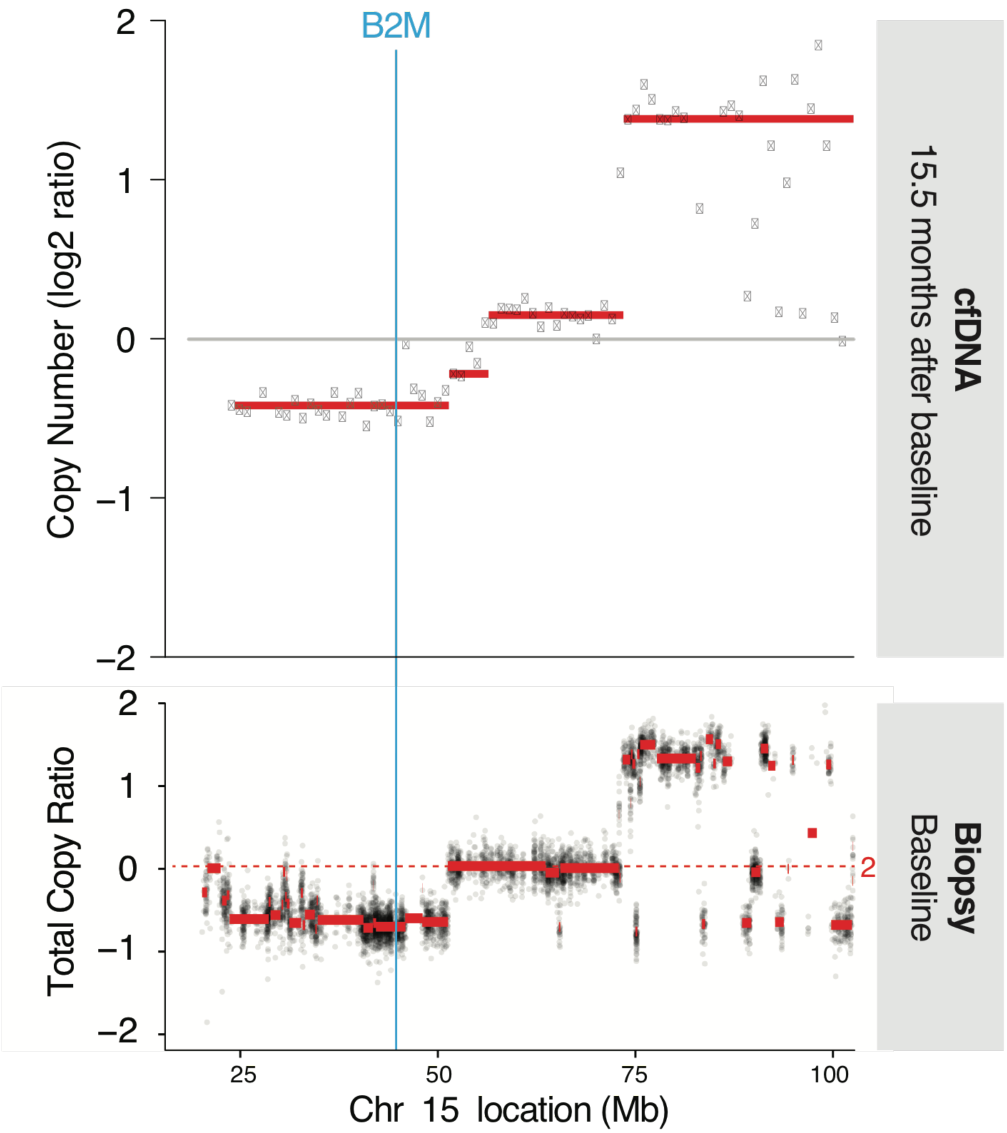

b Pat208

Months after baseline	Target	MUT events	WT events	Abundance (%)
24.3	NRAS p.Q61R	44	2508	1.7241
	B2M p.L13fs	17	1281	1.310

Supplementary Figure 15 | Detection of *B2M* mutation and LOH in cfDNA. (**a**) cfDNA was isolated from a blood biopsy taken from Pat99 15.5 months after initiation of CPB therapy when the patient was progressing (see **Methods**). Results from ReCapSeg of cfDNA (upper panels) and baseline biopsy (lower panel) are shown after performing WES on cfDNA sample. The vertical axis indicates position on chromosome 15. The horizontal axis indicates the total copy number ratio of targets. Each dot indicates a target region used to calculate copy ratio information. Solid red horizontal lines are segments inferred by the ReCapSeg algorithm. Dashed red lines indicate copy ratio corresponding to a total copy number of two, as inferred by ABSOLUTE. (**b**) cfDNA was isolated from a blood biopsy from Pat208 24.3 months after the baseline sample was taken. Results show the abundances of both *NRAS* p.Q61R mutation (used as positive control) and *B2M* p.L13fs, using specific probes and ddPCR (described in **Methods**).

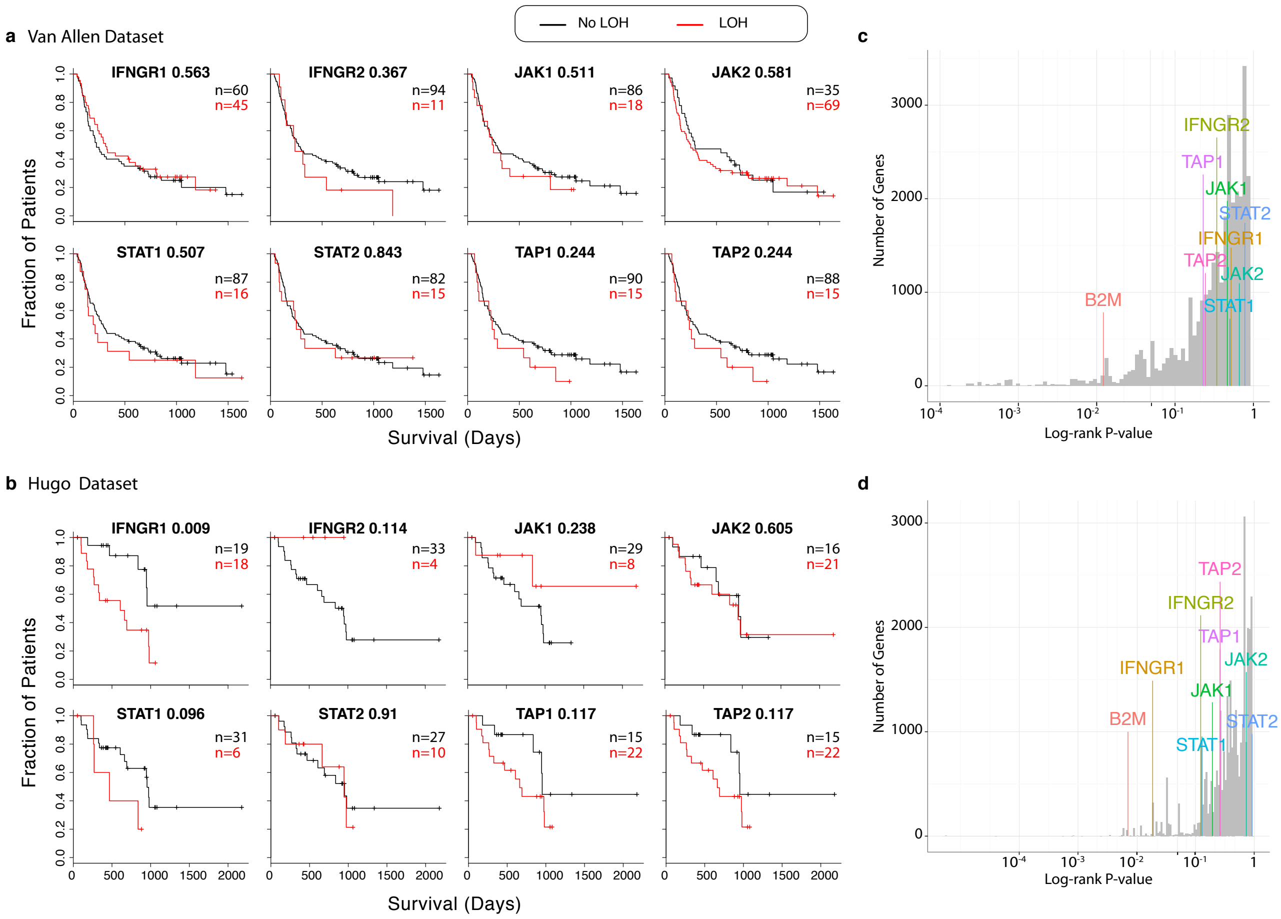

Supplementary Figure 16 | Clinical relevance of LOH in genes related to the interferon-gamma and antigen presentation pathways in two independent cohorts. Kaplan-Meier curves for patients with (red) and without (black) LOH in genes related to the interferon-gamma and antigen presentation machinery in the Van Allen dataset (**a**) or the Hugo dataset (**b**). Log-rank p-values are shown next to gene names in each subplot title. The numbers of patients with and without LOH are indicated. (**c-d**) A summary showing histograms for the log-rank p-value of genes with LOH that are related to the interferon-gamma and antigen presentation pathways within the Van Allen dataset (**c**) and Hugo data set (**d**). colored bars indicate the p-values that were obtained for the respective genes, heights are randomly staggered to enable the reading of the gene names.

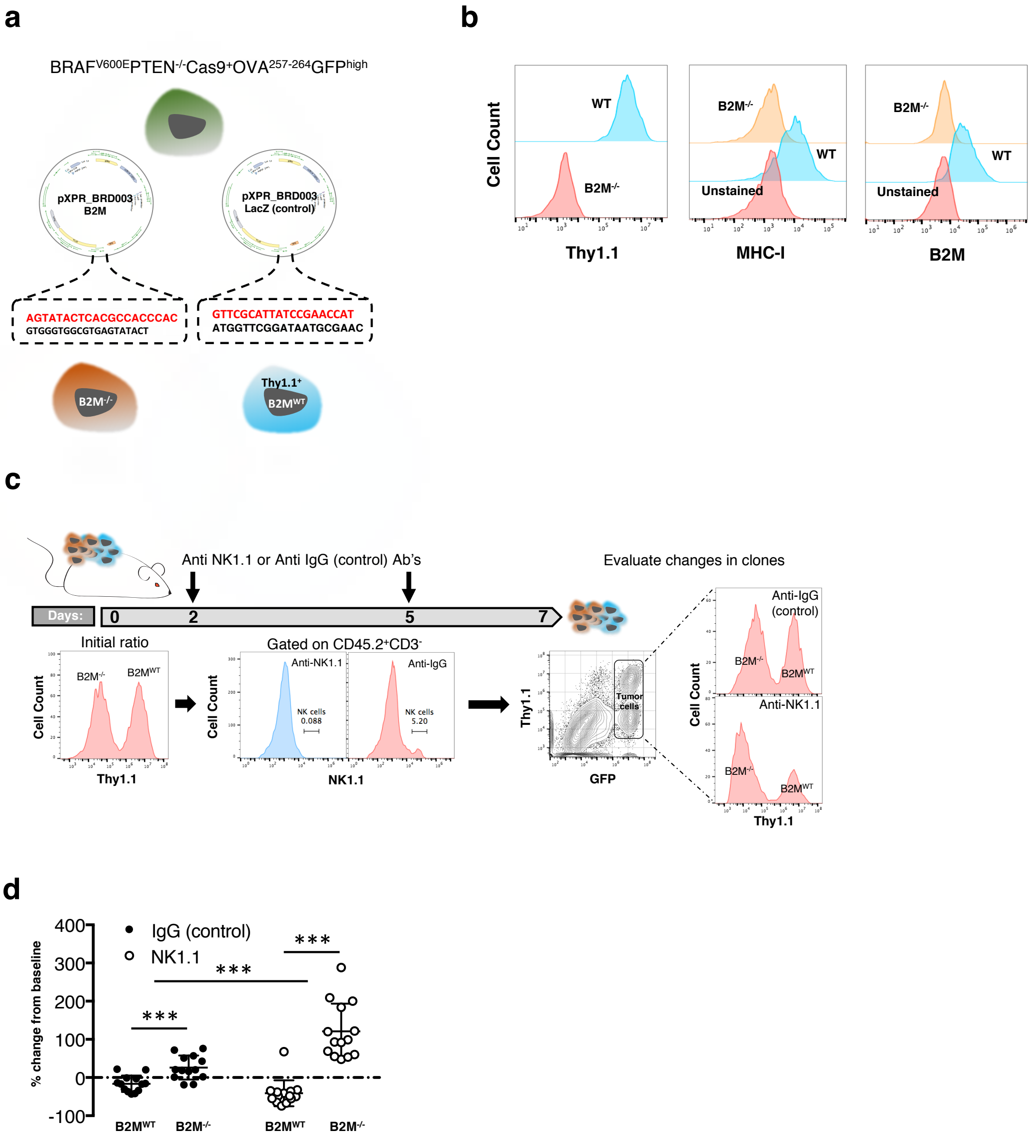
a
BRAFV600EPTEN-/-Cas9+OVA257-264GFPhigh
pXPR_BRD003 B2M
pXPR_BRD003 LacZ (control)
AGTATACTCACGCCACCCAC
GTGGGTGGCGTGAGTATACT
GTTCGCATTATCCGAACCAT
ATGGTTCGGATAATGCGAAC
B2M-/-
Thy1.1+
B2MWT
b
Cell Count
WT
B2M-/-
Thy1.1
Unstained
MHC-I
B2M
c
Anti NK1.1 or Anti IgG (control) Ab's
Evaluate changes in clones
Days:
0
2
5
7
Initial ratio
Gated on CD45.2+CD3-
Anti-NK1.1
Anti-IgG
NK cells
0.088
5.20
NK1.1
Thy1.1
GFP
Tumor cells
Anti-IgG (control)
d
% change from baseline
IgG (control)
NK1.1
***
400
300
200
100
0
-100
B2MWT
B2M-/-

Supplementary Figure 17 | NK cells control the expansion of melanoma tumor cells lacking *B2M*. (**a**) A schematic illustration showing the vectors and sequence of the guides used to generate the two cell lines clones: $BP^{Cas9+GFP+\mathit{B2M-/-}}$ and $BP^{Cas9+GFP+Thy1.1+\mathit{B2M+/+}}$ (described in **Methods**). (**b**) Evaluation of the total B2M and cell surface expression of Thy1.1 and MHC-I expression by flow cytometry after infecting the cells with the different lentivirus vectors. (**c**) Schematic illustration of the experimental procedure. $BP^{Cas9+GFP+\mathit{B2M-/-}}$ and $BP^{Cas9+GFP+Thy1.1+\mathit{B2M+/+}}$ clones were mixed in a 1:1 ratio and injected into the right flank of mice. On day +2 and +5 post transplantation one group of mice was treated with anti-NK1.1 to deplete NK cells. On day +7 mice were sacrificed, tumors were dissociated and the ratio between the two clones was evaluated with flow cytometry by gating on $GFP^+Thy1.1^+$ ($\mathit{B2M}^{+/+}$) or $GFP^+Thy1.1^-$ ($\mathit{B2M}^{-/-}$) cells (described in **methods**). (**d**) The percentage change for each clone from baseline was evaluated in the presence or absence of NK cells. Changes in frequencies were normalized to the baseline ratio of each clone. Results are representative of three independent experiments (error bars show SD, n=5 per group). ***p<0.0005 (t-test).
