# Supplementary material for: Resistance to checkpoint blockade therapy through inactivation of antigen presentation
Source: Nat Commun. 2017 Oct 26;8:1136. doi: 10.1038/s41467-017-01062-w (PMC5656607; doi:10.1038/s41467-017-01062-w)
Supplement: Supplementary file 3 — Supplementary Files [file 41467_2017_1062_MOESM3_ESM.pdf]

Description of Additional Supplementary Files

File Name: Supplementary Data 1
Description: Patients clinical parameters.

File Name: Supplementary Data 2
Description: List of all non-silent mutations found in all six biopsies taken from Pat208, who was treated with CPB and developed resistance after an initial response.

File Name: Supplementary Data 3
Description: Gene sets used to evaluate the presence of different immune cell types, immune cell state and tumor microenvironment factors from the RNAseq data.

File Name: Supplementary Data 4
Description: Summary of the mutations and LOH in genes related to the IFNγ and antigen presentation machinery found in the three analyzed cohorts.

File Name: Supplementary Data 5
Description: IHC protocols summary.
