# Supplementary material for: Resistance to checkpoint blockade therapy through inactivation of antigen presentation
Source: Nat Commun. 2017 Oct 26;8:1136. doi: 10.1038/s41467-017-01062-w (PMC5656607; doi:10.1038/s41467-017-01062-w)
Supplement: Supplementary file 2 — Peer Review File [file 41467_2017_1062_MOESM2_ESM.pdf]

Editorial Note: This manuscript has been previously reviewed at another journal that is not operating a transparent peer review scheme. The manuscript was considered suitable for publication without further review at Nature Communications.
